# Projected Increase in Hydropower Production in India under Climate Change

**DOI:** 10.1038/s41598-018-30489-4

**Published:** 2018-08-20

**Authors:** Syed Azhar Ali, Saran Aadhar, Harsh L. Shah, Vimal Mishra

**Affiliations:** Civil Engineering, Indian Institute of Technology Gandhinagar, Gandhinagar, 382355 India

## Abstract

Hydropower is a valuable renewable energy resource in India, which can help in climate change mitigation and meet the increasing energy demands. However, the crucial role of climate change on hydropower production in India remains unexplored. Here using the observations and model simulations, we show that seven large hydropower projects experienced a significant (p-value < 0.05) warming and a decline in precipitation and streamflow during the observed period of 1951–2007. However, all the hydropower projects are projected to experience a warmer and wetter climate in the future. Multimodel ensemble mean annual average temperature (precipitation) is projected to rise up to 6.3 ± 1.6 °C (18 ± 14.6%) in the catchments upstream of the other reservoirs by the end of the 21st century under representative concentration pathway (RCP) 8.5. Due to the projected increase in precipitation, mean annual streamflow (up to +45%) and hydropower (up to +25%) production are projected to rise under the future climate. However, significant warming (6.25 ± 1.62 °C) is projected to result in a decline in streamflow and hydropower production in May- June for snow-dominated Nathpa Jhakri and Bhakra Nangal hydropower projects. Our results provide insights into the development and planning of hydropower projects in India under the current projected future climate.

## Introduction

India is the 7^th^ largest hydroelectric power producer in the world and has a high potential for hydropower generation. Hydropower is the second largest contributor of energy consumed in the Indian power sector^1^. India has utilized only 17% of the total 15,000 MW hydropower potential and tremendous opportunities for future expansion exist^2^. The most significant hydropower potential in India exists in the three major transboundary river basins (Ganges, Indus, and the Brahmaputra)^2^. However, all these basins have experienced substantial changes in precipitation and air temperature that affected water availability for hydropower generation^3^.

Since hydropower production and its potential depends on streamflow, it is sensitive to climate change^4^. The impacts of climate change on hydropower potential have been studied globally. For instance, Liu *et al*.^5^ reported that Gross Hydropower Potential (GHP), which is total hydropower generation from all natural runoff at the outlet of a specific region, of China is projected to change by −1.7 to 2% in the near future (2020–2050) and 3 to 6% by the late 21^st^ century (2070–2099). Moreover, they^5^ found that annual Developed Hydropower Potential (DHP), which is the maximum possible production of hydropower at the existing hydroelectric facilities, is projected to decline by 2.2 to 5.4% from 2020 to 2050 and 1.3 to 4% from 2070 to 2099. Turner *et al*.^6^ showed approximately ±5% change in mean global hydropower production by 2080 s. There are uncertainties in the projections of annual hydropower production. For instance, global theoretical hydropower potential (THP), the maximum hydropower production under the ideal condition without any losses, is projected to increase moderately due to climate change^7^. However, Van Vliet *et al*.^8^ reported reductions in global annual hydropower capacities of 0.4 to 6.1% by 2080 s.

India has experienced significant warming over the past few decades^9^, which is likely to continue along with the changes in precipitation in the 21^st^ century^10^. Despite the profound implications of climate change on streamflow, the linkage between climate change and hydropower production in India remains unexplored. Here, we provide the first-ever assessment of climate change impacts on hydropower potential of the seven large hydropower projects with more than 300 MW installed capacity in India. These reservoirs are of the national importance, and most of them fall among the top 10 hydropower projects in India.

## Results and Discussion

**Figure 1:**
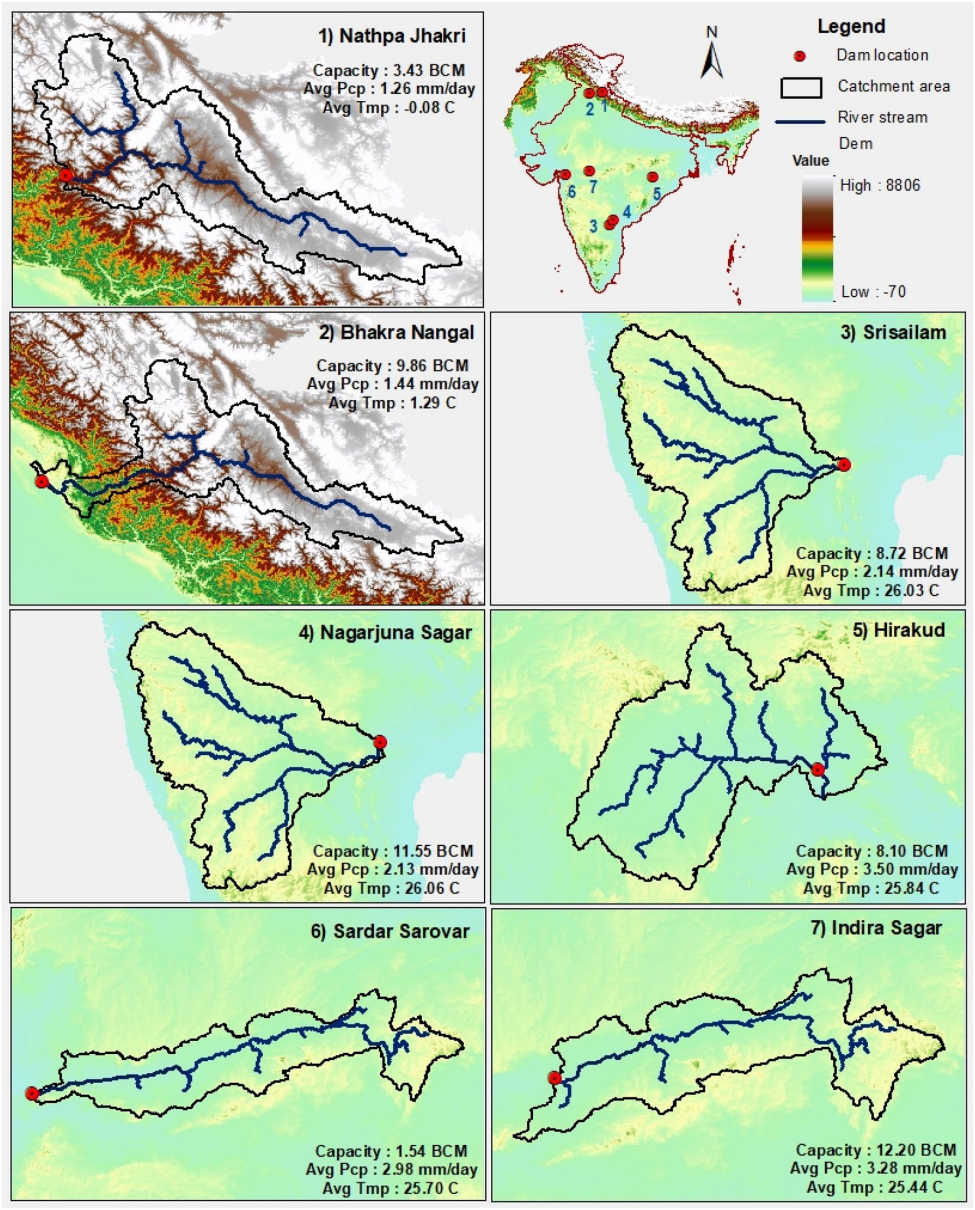
Location of the seven large dams in India along with their topography, upstream catchment area, stream network, reservoir storage capacity, and average annual precipitation for (1) Nathpa Jhakri, (2) Bhakra Nangal, (3) Srisailam, (4) Nagarjuna Sagar, (5) Hirakud, (6) Sardar Sarovar, and (7) Indira Sagar. For more details, please see supplemental Tables S1–S2. The figure was developed using the Generic Mapping Tools (GMT) version 5.4.2 (http://gmt.soest.hawaii.edu).

We performed the analysis on seven (Nathpa Jhakri, Bhakra Nangal, Srisailam, Nagarjuna Sagar, Hirakud, Sardar Sarovar, and Indira Sagar) large hydropower projects in India (Fig. 1, and Table S1). These large reservoirs are located in four major Indian sub-continental basins: Indus, Krishna, Mahanadi, and Narmada (Tables S1, S2). Nathpa Jhakri and Bhakra Nangal reservoirs are located on Satluj River, which flows from the snow dominated upper part of the Indus basin (Fig. 1). Satluj River has the third largest drainage area in Himalaya and elevation in Satluj river varies from 400 to 7200 m. About 66% of the drainage area of Satluj River falls in China, and the rest is in India. At higher elevations in Satluj basin, snowfall occurs during December to March while summer (July to September) is monsoon dominated. Mean annual precipitation is 1.2 and 1.4 mm/day while mean annual air temperature in Nathpa Jhakri and Bhakra Nangal is −0.08 and 1.3 °C, respectively (Fig. 1). Mean annual glacier contribution at Bhakra dam on Satluj River is about 4.8%^11^. The other five reservoirs are located in the monsoon-dominated climate in central-south India (Fig. 1, Table S1). Mean annual precipitation in the five reservoirs located in central-south India varies from 2.1 to 3.50 mm/day while mean annual air temperature ranges from 25.4 to 26.0 °C (Fig. 1). The total installed capacity of Srisailam, Nathpa Jhakri, Sardar Sarovar, Bhakra Nangal, Indira Sagar, Nagarjuna Sagar, and Hirakud are 1670, 1500, 1450, 1325, 1000, 816, and 307.5 MW, respectively.

**Figure 2:**
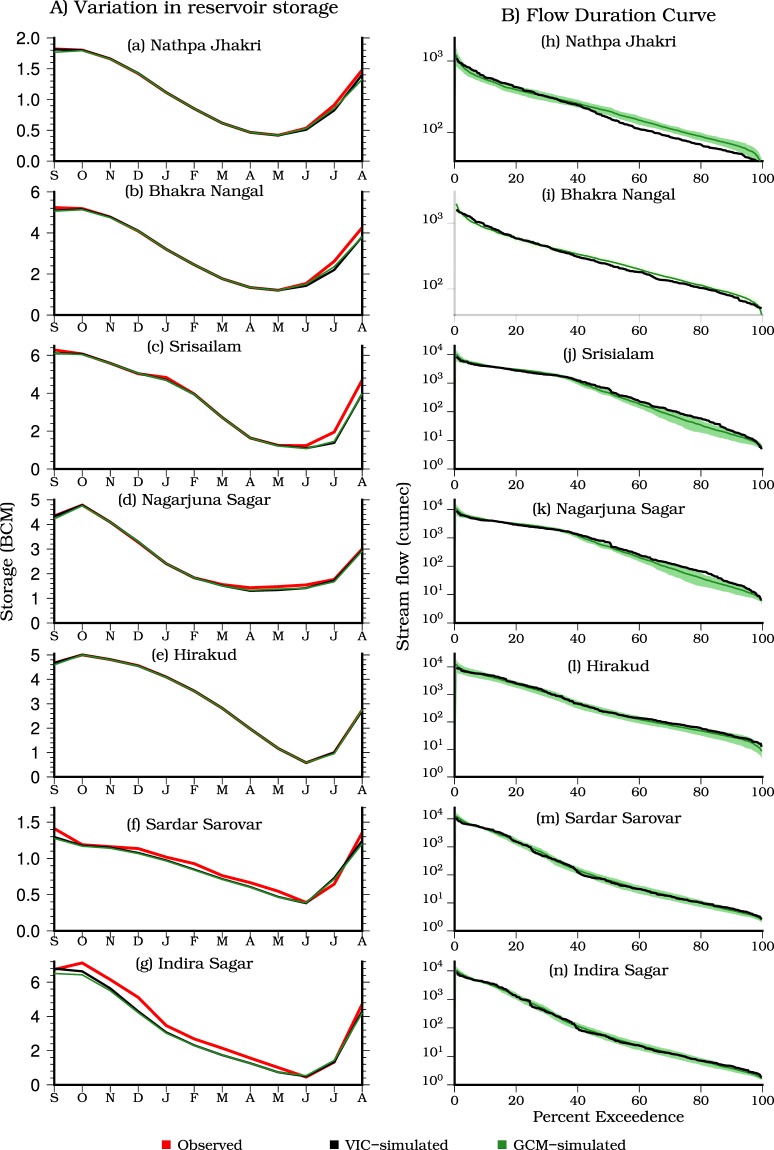
**(A)** Comparison of observed storage reservoir storage (red) (BCM) with the VIC model simulated reservoir storage (black) with the observed meteorological forcing and multimodel ensemble mean storage (green) simulated using the VIC model using downscaled and bias-corrected forcing for 10 CMIP5-GCMs for the period of 2002–2014. **(B)** Comparison of flow duration curves obtained using the VIC model simulated streamflow with observed data (black) and downscaled and bias-corrected data from the GCM models (green) for the historic period (1971–2000). Shaded regions show intermodel variability for the historic (green) periods. The figure was developed using the Generic Mapping Tools (GMT) version 5.4.2 (http://gmt.soest.hawaii.edu).

We evaluated the performance of reservoir mechanism (hereafter inflow to the reservoir is mentioned as streamflow)^12^ by comparing the observed and the VIC model simulated seasonal variation in reservoir storage (Fig. 2A). The VIC model was calibrated and evaluated for monthly streamflow (see methods for details). The VIC model simulated reservoir storage compares well against the observed reservoir storage. The VIC simulated reservoir storage for the historic (1971–2000) period from Coupled Model Intercomparison Project Phase 5 (CMIP5)-GCMs is in a good agreement with the observed storage, which indicates the effectiveness of the bias-correction method (Fig. 2).

**Figure 3:**
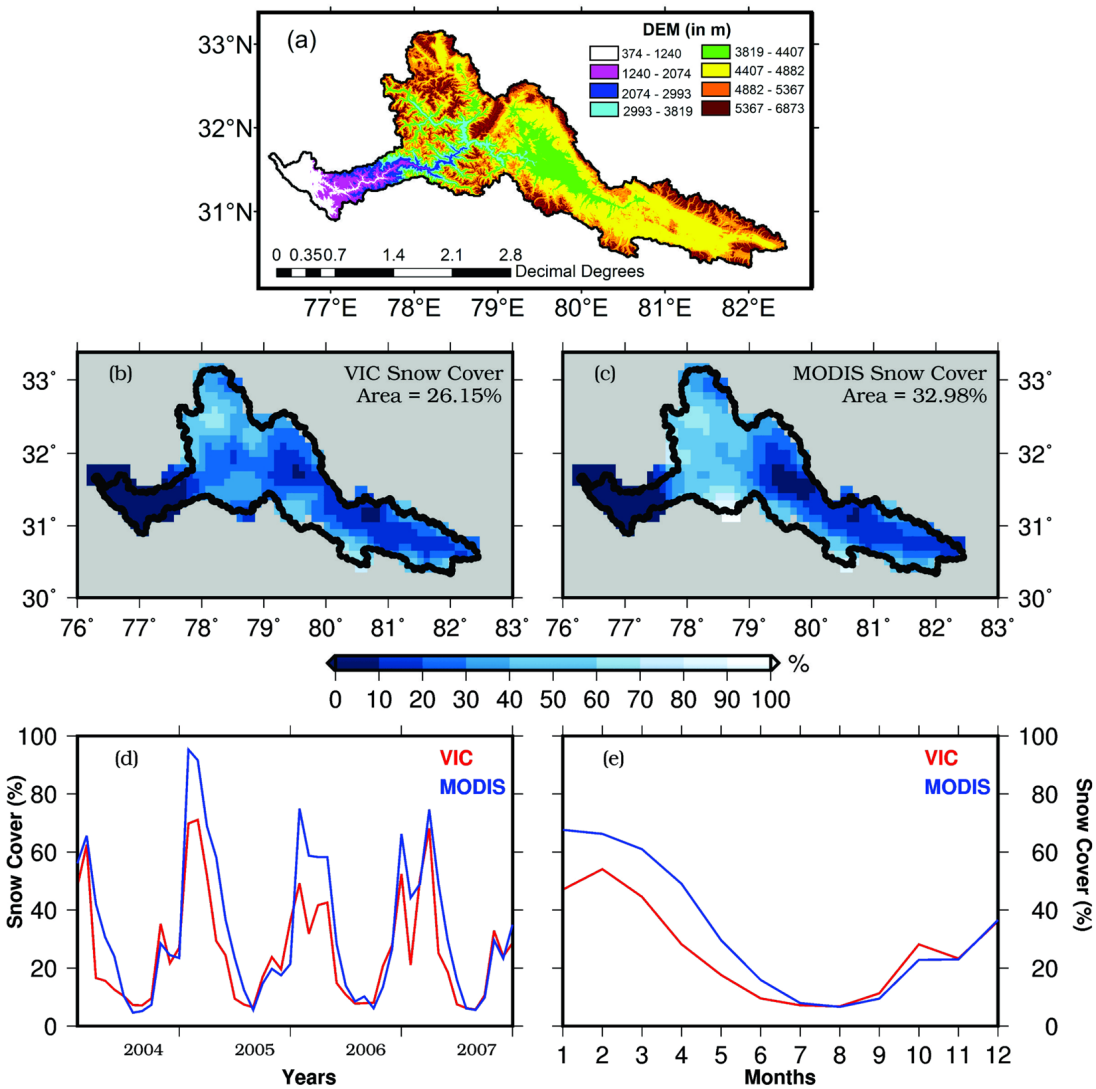
**(a)** Digital elevation model for Satluj River basin in which Nathapa Jhakri and Bhakhara Nangal reservoirs are located. **(b–e)** Comparison of snow cover obtained using the VIC model (red) with MODIS (blue) for the period of 2004–2007. The figure was developed using the Generic Mapping Tools (GMT) version 5.4.2 (http://gmt.soest.hawaii.edu).

Flow duration curves, which describe the relationship between the probability of exceedance of time and flow magnitude^13–15^, are used to estimate DHP in the observed and projected future climate. The VIC model simulated flow duration curves for the observed and historic (from CMIP5-GCMs) reference period compare well against the observed flow duration curves (Fig. 2B). Apart from the VIC model performance against observed streamflow (Fig. S1), we evaluated VIC simulations against snow cover^16^ obtained from Moderate Resolution Spectroradiometer (MODIS- Terra), which is available at 0.05° spatial and monthly temporal resolutions (Fig. 3). The VIC simulated snow cover captures observed monthly and seasonal variability (in MODIS based snow cover) reasonably well during the 2004–2007, however, underestimates the snow-covered area (Fig. 3). For instance, the average snow-covered area from the VIC simulations and MODIS are 26.1% and 32.9%, respectively for 2004–2007. We attribute this underestimation of the snow covered area to the quality of meteorological forcing. The density of gage stations in the Himalayan region is less, which can cause uncertainty in observations of temperature and precipitation^17^. Since snow simulations of the VIC model are sensitive to temperature and precipitation^18^, errors and uncertainty in meteorological forcing^17,19,20^ can cause bias in the snow-covered area. Bookhagen and Burbank^19^ reported that there is strong spatial variability in precipitation in the Himalayan region, which, however, cannot be well captured with the limited number of gage stations. Overall, the performance of the VIC model to simulate streamflow, snow cover, reservoir storage, and flow duration curves provides a basis for the assessment of DHP under the projected future climate for these major reservoirs.

Next, we analyzed the changes in the observed mean annual precipitation and air temperature for the period of 1951–2007 (Table S3). We find that most of the catchments (except Nathpa Jhakri and Bhakra Nangal) upstream of these large reservoirs experienced a significant (p-value < 0.05) warming and a non-significant (p-value > 0.05) decline in mean annual precipitation during the observed period (Table S3). For all the reservoirs, mean annual streamflow has declined from 1951 to 2007 (Fig. S2, Table S3). Consistent with the findings of Palazzi *et al*. (2012)^21^, we find a significant (p-value < 0.05) decline in mean annual precipitation and a moderate increase (p-value > 0.05) in mean annual temperature resulted in significant (p-value < 0.05) reduction in streamflow at Nathpa Jhakri and Bhakra Nangal reservoirs (Table S3, Fig. S2). Other five reservoirs show a moderate but not significant decline in mean annual streamflow during the period 1951–2007 (Fig. S2, Table S3). Overall, we find that all the large hydropower projects experienced a dry and warm climate that resulted in a decline in mean annual streamflow during the period of 1951–2007.

Year-to-year variability, estimated using the coefficient of variation (CV) of streamflow, ranges between 0.30 and 0.65 for all the reservoirs with higher values for Srisailam and Nagarjuna Sagar (Fig. S3a). We analyzed composite anomalies of streamflow (%) for the top five dry, and wet years from 1951 to 2007 (Table S4). Streamflow during the monsoon season for the five driest years (Table S4) on the record (1951–2007) shows a significant deficit (more than 40%) indicating that prolonged droughts have a significant influence on streamflow and hydropower production (Fig. S3b). Similarly, during the top five wet years (Table S4), the monsoon season streamflow was higher than 60% of its long-term mean for most of the reservoirs (Fig. S3c). Our results highlight the potential role of observed climate variability and climate extremes (dry and wet years) on streamflow and hydropower production.

Next, we estimated projected changes in precipitation, temperature, streamflow (VIC simulated), and DHP using bias-corrected climate projections (see methods for details) for the seven reservoirs in India (Table S5). We considered the two representative concentration pathways (RCPs: 2.6 and 8.5) for the analysis and changes were estimated for the near (2010–2039), mid (2040–2069), and end (2070–2099) terms of the 21^st^ century to the historical reference (1971–2000) period. The RCP 2.6 represents the low warming scenario while RCP 8.5 represents the high emission scenario leading to a higher rise in the global mean temperature by the end of 21^st^ century^22,23^.

Most of the catchments of the large reservoirs are projected to experience a substantial warming under the projected future climate. Consistent with previous studies^24–26^, the highest warming is likely for Nathpa Jhakri and Bhakra Nangal where the annual mean temperature is projected to increase by more than 6.25 ± 1.5 °C by the end (2070–2099) of the 21^st^ century under the RCP 8.5. Multimodel ensemble mean annual average temperature for the future period is projected to rise between 1.38 and 6.32 °C in the catchments upstream of the other reservoirs (Table S5). All the seven reservoirs are projected to experience a wetter (up to 18% increase) scenario in the projected future climate. Under the RCP 8.5, Nathpa Jhakri and Bhakra Nangal are projected to experience 17.04 ± 20.76% and 15.30 ± 20.16% increase in mean annual precipitation by the end of 21^st^ century (Table S5). The projected increase in precipitation under the warming climate in the Himalayan catchment is consistent with previous studies^21,27^. However, our results differ from the findings of Su *et al*.^28^, who reported a 60% increase in the monsoon season precipitation in the late 21^st^ century in the upper Indus river basin. Their^28^ findings are based on the climate model outputs that were not bias corrected. The projected increase in mean annual precipitation for Srisailam and Nagarjuna Sagar, Hirakud, Sardar Sarovar, and Indira Sagar is 18.3 ± 14.6% and 18.5 ± 14.7%, 13.2 ± 14.3%, 17.7 ± 17.77%, and 15.75 ± 17.64%, respectively in the late 21^st^ century under the RCP 8.5.

**Figure 4:**
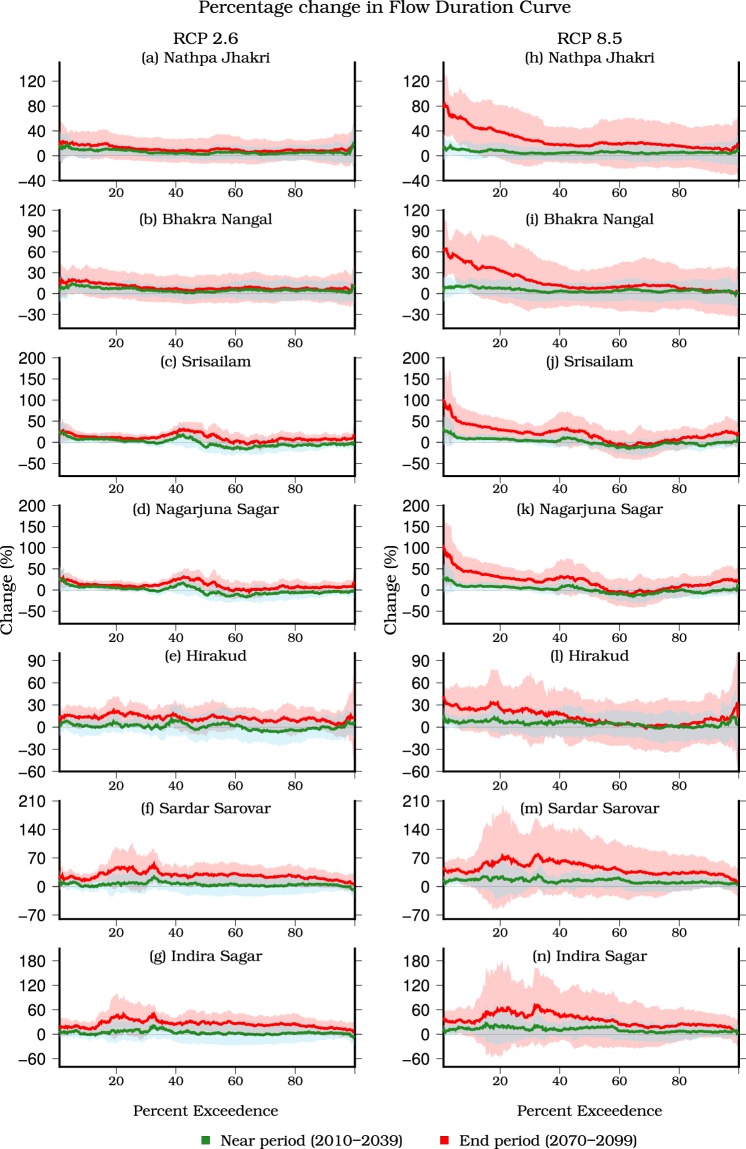
Multimodel ensemble mean change in flow duration curves for the near (2010–2039, green) and end (2070–2099, red) period for the RCP 2.6 and RCP 8.5. Changes were estimated for each CMIP5-GCM with respect to the historic reference (1971–2000) period and then ensemble mean was estimated. The shaded region shows intermodel variation estimated using one standard deviation for the near (cyan) and end (pink) period. The figure was developed using the Generic Mapping Tools (GMT) version 5.4.2 (http://gmt.soest.hawaii.edu).

We estimated the multimodel ensemble mean changes in the flow duration curves and streamflow under the projected future climate (Fig. 4). While the intermodel uncertainty in changes is substantial (Fig. 4), we find that the flow duration curves for Bhakra Nangal and Nathpa Jhakri show a negligible change in flow under the RCP 2.6 (Fig. 4a,b). In contrast to the low warming scenario (RCP 2.6), flow is projected to increase for Bhakra Nangal and Nathpa Jhakri under the RCP 8.5 (Fig. 4h,i). High flow (probability of exceedance in time less than 10%) is projected to increase substantially in all the reservoirs at the end of the 21^st^ century in RCP 8.5 scenario (Fig. 4). Moreover, mean annual streamflow is projected to increase for all the reservoirs by the end of the 21^st^ century (Table S5, Figs S4–S6). The projected increase in streamflow varies between 25.30 ± 22.7% (Hirakund) and 44.75 ± 35.9% (Nagarjuna Sagar) at the end of the 21^st^ century under RCP 8.5 (Table S5).

**Figure 5:**
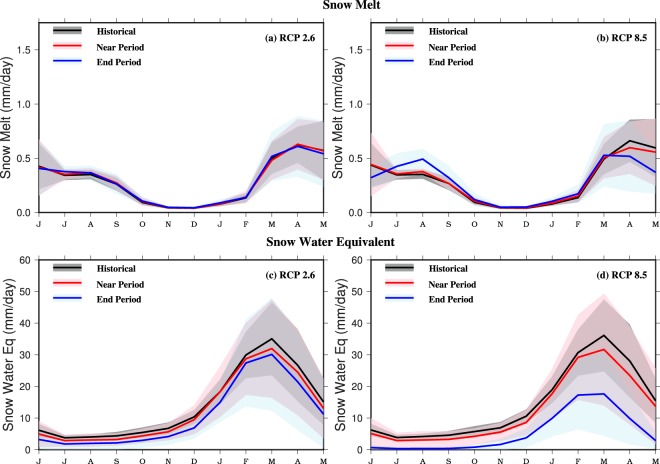
Area averaged multimodel ensemble mean snowmelt (mm) and snow water equivalent (mm) for the historic (1971–2000, black), near (2010–2039, red), and end period (2070–2099, blue) under RCP 2.6 and RCP 8.5 for Satluj River basin. Shaded regions show intermodel variation for 10 CMIP5-GCMs for historic, near, and end period. The figure was developed using the Generic Mapping Tools (GMT) version 5.4.2 (http://gmt.soest.hawaii.edu).

The projected increase in streamflow for the reservoirs located in the central and south India can be attributed to the increase in the monsoon season precipitation under the future climate^29,30^. Streamflow and water availability in India is largely dominated by the changes in the monsoon season precipitation^10,31^, and projected warming plays a secondary role. We find that snow cover and snow depth is projected to decline under the projected future climate in the catchment of Nathpa Jhakri and Bhakra Nangal (Figs S7 and S8). The projected decline in snow depth and snow cover will result in reduced snow water equivalent and early snowmelt (Fig. 5). Moreover, snowfall is projected to reduce substantially during the winter season by the end of the 21^st^ century under RCP 8.5 (Fig. S9) while rainfall is projected to increase during the monsoon season (Fig. S10). The projected decline in snowfall and snowmelt is consistent with the changes during the observed period. Bhutiyani *et al*.^32^ reported that in Satluj River basin, the contribution of glacier and snowmelt has declined during 1922–2004. A comparison between snow and rain suggests a more substantial increase in the monsoon season rainfall in contrast to the reduction in the amount of snow, which results in an overall increase in streamflow in the basin (Table S5).

**Figure 6:**
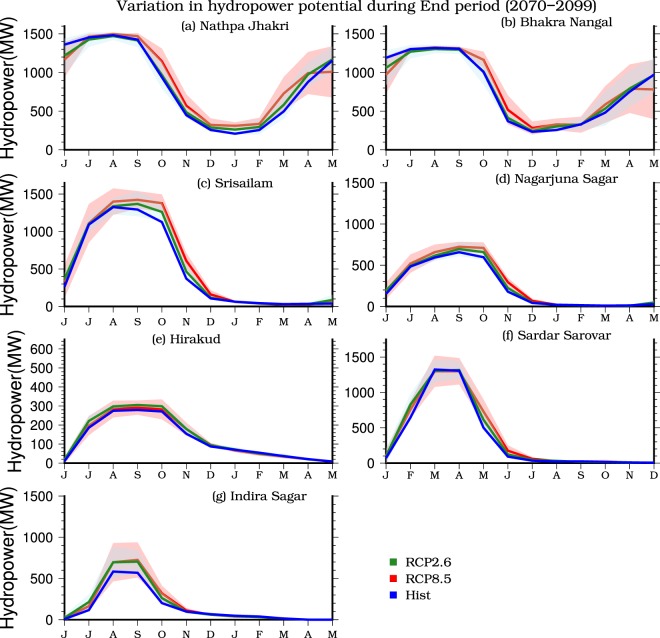
Seasonal cycle of multimodel ensemble mean developed hydropower potential (DHP, MW)for the historic (1971–2000, blue) and end period (2070–2099) periods under RCP 2.6 (green) and RCP 8.5 (red) for **(a)** Nathpa Jhakri, **(b)** Bhakra Nangal, **(c)** Srisailam, **(d)** Nagarjuna Sagar, **(e)** Hirakud, (**f**) Sardar Sarovar, and (**g**) Indira Sagar. Shaded regions show intermodel variation for 10CMIP5-GCMs under RCP 2.6 (cyan) and RCP 8.5 (pink). The figure was developed using the Generic Mapping Tools (GMT) version 5.4.2 (http://gmt.soest.hawaii.edu).

Finally, we estimated DHP using the VIC model simulated streamflow and other parameters for the reservoirs (see methods for details). DHP projections are in general similar to streamflow projections showing a projected increase under warming climate (Fig. 6). However, DHP is projected to decline in May-June for Bhakra Nangal and Nathpa Jhakri reservoirs mainly due to the projected decline in streamflow in summer (Fig. 6 and Table S5). This projected decline in streamflow during May-June can be attributed to a decrease in snow water equivalent and snowmelt in May-June under warming climate (Fig. 5). Multimodel ensemble mean projected changes in DHP are 0.68 ± 6.4%, 0.22 ± 6.90%, 2.41 ± 4.64%, 3.90 ± 6.62%, 0.42 ± 4.00%, 0.99 ± 5.86%, and 2.87 ± 12.45% for Nathpa Jhakri, Bhakra Nangal, Srisailam, Nagarjuna Sagar, Hirakud, Sardar Sarovar, and Indira Sagar, respectively in the near term under RCP 2.6 (Fig. S11 and Table S5). Under RCP 8.5, DHP is projected to increase for all the reservoirs and throughout the 21^st^ century (Figs 6, S11, and Table S5).

We find that the two reservoirs (Nathpa Jhakri and Bhakra Nangal) located in Northern India are projected to become precipitation dominated under the future climate. Changes in snow storage result in a projected decline in streamflow and DHP in May-June when most of the streamflow comes from snowmelt. Nathpa Jhakri and Bhakra Nangal reservoirs are projected to experience much higher warming (more than 6.25 °C) in comparison to the other reservoirs located in the monsoon-dominated climate. Significant warming results in a decline in snow storage, causing a reduction in streamflow in May-June, which is consistent with the findings of the previous studies^10,26,33^. The reservoirs located in central and southern India are projected to experience an increase in streamflow and DHP, which is mainly because of an increase in the monsoon season precipitation under the projected future climate^29,30,34^. Despite the large uncertainties in the summer monsoon rainfall projections^35^, a majority of GCMs project an increase in the future climate^29,36^. However, our results show a substantially less increase (less than 18.5%) in the projected future climate as reported by Menon *et al*.^29^. As in most of the sub-continental river basins, streamflow is dominated by the changes in the summer monsoon precipitation^10^. For the two large reservoirs located in northern India, snowfall is projected to decline while rainfall (monsoon season) is projected to increase causing an overall increase in DHP.

While the uncertainty in climate projections is important, other factors can also influence projections of streamflow and DHP. For instance, land use and land cover changes are likely to occur in future in response to urbanization and expansion of agriculture^37^ that will affect streamflow^38,39^ and so as hydropower production. The projected increase in precipitation and streamflow may also result in an increased sediment load to the reservoirs^40,41^, which can affect the storage capacity of reservoirs and hydropower potential in future. Moreover, future irrigation demands can also influence the hydropower potential of the reservoirs. For instance, Zeng *et al*.^42^ based on a sensitivity analysis reported that reservoirs in India are likely to experience reduced hydropower production if irrigation demands increase in future. However, Raje and Mujumdar^43^ found that without compromising the role of reservoirs for flood control and irrigation, hydropower generation can be increased by climate change. Apart from these, the contribution of glaciers to streamflow can play a major role in the projected changes in hydropower of Nathpa Jhakri and Bhakra Nangal reservoirs. Present contribution of glaciers in mean annual streamflow for these reservoirs is relatively lower (~5%)^11^. However, an explicit representation of glaciers and other processes mentioned above with better calibration of the VIC model can improve our understanding of the projected changes in streamflow and hydropower for these two reservoirs. Notwithstanding these limitations, our results provide important insights on the impacts of observed climate variability and projected future climate change on streamflow and hydropower production in India, which can assist planners and policy-makers. However, for future planning, careful consideration of uncertainties in precipitation projections along with robust and comprehensive adaptation strategies are required^33^.

## Methods

### Observed and projected climate forcing

Long-term daily precipitation, air temperature (maximum and minimum), and wind speed were used as meteorological forcing to run the Variable Infiltration Capacity (VIC) model. The observed daily precipitation and minimum and maximum temperatures data (0.25° spatial resolution) were obtained from the India Meteorological Department (IMD)^44^. The observed data from IMD have been used in Mishra and Lilhare^10^, Shah and Mishra^3^, and Mishra *et al*.^45,46^. For the study domain that falls outside India (part of Bhakra Nangal Catchment), observed daily precipitation data were obtained from APHRODITE^17^ at 0.25° spatial resolution. Maximum and minimum temperatures and wind speed data were obtained from Sheffield *et al*.^47^ at 0.25^o^ resolution for the 1951–2007 period. Recently, Aadhar and Mishra^48^ used APHRODITE and meteorological data^47^ to analyze drought over South Asia. We used MODIS^49^ monthly snow cover data at 0.05° to evaluate the VIC model for the period 2004–2007.

To understand the impacts of the projected future climate on streamflow and DHP, we forced the VIC model with bias-corrected daily precipitation and temperature (maximum and minimum) obtained from 10 CMIP5-GCMs^50^ (Table S6). We selected the 10 GCMs (GFDL-CM3, GFDL-ESM2G, GFDL-ESM2M, MIROC5, MIROC-ESM, MIROC-ESM-CHEM, NorESM1-M, CCSM4, CESM1-CAM5, and HadGEM2-AO) out of about 40 CMIP5 models. The daily data from GCMs were bias corrected for the 1951–2099 period [historic: 1951–2005 and future: 2006–2099 periods] using the methodology of Hempel *et al*.^51^, which is based on trend-preserving statistical bias correction developed within the Inter-Sectoral Impact Model Intercomparison Project (ISIMIP). Bias correction was performed using observed precipitation and temperature from IMD for the Indian region and data from APHRODITE^17^ and Sheffield *et al*.^47^ for outside India. Similar approaches, based on bias-corrected data, are used to assess projected changes in hydropower^52^.

Streamflow and DHP analysis for the future climate were carried out using the VIC simulated streamflow for the near (2010–2039), mid (2040–2069), and end (2070–2099) periods. Projected changes in the future climate were estimated against the historical reference period of 1971–2000. Since our assessment for the projected future climate is based on bias-corrected data from the CMIP5-GCMs, we first evaluated the effectiveness of the bias-corrected data against the observations. We find that all-India averaged seasonal cycle of the bias-corrected GCMs precipitation and temperature were well compared with the observed precipitation and temperature for the historic reference period (1971–2000) (Fig. S13a,b).

### The Variable Infiltration Capacity (VIC) model

We used the VIC model to simulate streamflow in the observed and projected future climate. The VIC model^53^ is a semi-distributed and physically based hydrological model that solves both water and energy balance for each grid-cell. The VIC model uses the infiltration mechanism utilized in the Xinanjiang model^54^ to generate runoff from precipitation higher than the available infiltration capacity. Baseflow in the VIC model is computed using Arno model conceptualization^55^. Snow model^56^ and frozen soil algorithm^18,57,58^ in the VIC model are used to calculate cold season processes (snow storage and melt). In the snow model, a two-layer snowpack is represented, which accounts for refreezing of meltwater, the role of vegetation, and snow age-dependent albedo^56^. We did not explicitly represent glaciers in the VIC model. However, we followed the approach of Schaner *et al*.^11^ to account for the snow and glaciers on hydrological processes. We used a 57 year (1951–2007) spin up period for the VIC model to initialize the soil water storage and to create snow reservoirs (similar to glaciers) in the high elevation areas. For each 0.25° grid, we used ten snow bands to consider elevation variation within the grid cell. More information on the snow model and glacier simulations can be obtained from Cherkauer *et al*.^18^ and Schaner *et al*.^11^, respectively.

The University of Maryland global vegetation classifications (UMD GLCF), available at 1 km spatial resolution^59^ was used to develop vegetation parameters. Soil parameters were derived from the Food and Agriculture Organization^60^ dataset available at 5-minute spatial resolution. The parameters used for the calibration of the VIC model include infiltration parameter (Binf), soil layers (second and third layer) thickness (D), maximum baseflow velocity (Dsmax), the fraction of maximum baseflow velocity (Ds), and the fraction of maximum soil moisture content (Ws). The simulated runoff and baseflow from each grid-cell were routed using a standalone routing model^61,62^, which explicitly represents the routing of surface and subsurface runoff within a grid using a unit hydrograph that contributes to a channel network. The VIC model has been applied in the Indian sub-continent in several previous studies. For instance, daily streamflow simulations using the VIC model were used to examine the bias and uncertainty in satellite-based precipitation products over the Indian subcontinental river basins for the period of 2000–2013^3^, while Shah and Mishra^9^ evaluated hydrologic changes in the Indian sub-continental river basins using the VIC model. In our simulations, the VIC model does not consider dynamic vegetation for the observed and future climate, sediment load in streamflow, explicit representation of glaciers, and human influence on the water cycle (e.g., groundwater abstraction, reservoir operation). These factors can affect streamflow and reservoir capacity. Despite these limitations, the streamflow changes in the natural water cycle are useful to evaluate the changes in the hydropower during the warming climate.

### Calibration and evaluation of the VIC model

The observed streamflow data were obtained from the India Water Resources Information System (WRIS) and the Global River discharge database-SAGE. Simulated monthly streamflow from the VIC model was compared with the observed monthly streamflow at the gage location to evaluate the ability of the model (Fig. S1). Monthly streamflow was calibrated at the gage stations that are located upstream of the reservoirs (Table S2). Therefore, these stations are not substantially affected by abstraction^9^. Nash Sutcliffe Efficiency^63^ (NSE) and correlation coefficient (r) between observed and simulated streamflow were used to evaluate the performance of the model (Table S7). All the four basins showed NSE greater than 0.7 and correlation coefficient (r) greater than 0.85 during the calibration periods (Table S7). The performance of the VIC model was satisfactory for the evaluation periods as well (Fig. S1 and Table S7). The performance of hydrologic models during the observed period can reduce the uncertainty in climate change impacts assessment^64^. However, since our analysis is focused on the projected changes (with respect to the historical), the performance of the hydrologic model may not be crucial.

### Estimation of hydropower potential

Developed hydropower potential (DHP) of the seven large reservoirs was estimated based on monthly release using generic regulation rules as described in Hanasaki *et al*.^12^. DHP is the maximum hydropower generation based on the current hydroelectric facilities using the existing information of the reservoir such as Installed Hydropower Capacity (IHC), storage capacity, and dam height^5^. The monthly release contains the effect of annual and monthly variability in the inflow to the reservoirs. When the storage capacity is large as compared to the mean annual inflow to the reservoir, the monthly release is not influenced by the monthly inflow and remains uniform over the year. On the other hand, when the storage capacity is in the order of annual inflow, the monthly release fluctuates every month. The monthly release R_m_ (m^3^/s) from a reservoir was estimated as the case of no irrigation demands as described in Hanasaki *et al*.^12^:

1 $${{\rm{R}}}_{{\rm{m}}}=\{\begin{array}{ll}{k}_{y}{i}_{a}, & {\rm{c}}\ge 0.5\\ {(\frac{c}{0.5})}^{2}{k}_{y}{i}_{a}+\{1-{(\frac{c}{0.5})}^{2}\}{i}_{m} & 0 < {\rm{c}} < 0.5\end{array}$$

where i_m_ is monthly inflow (m^3^/s), i_a_ is mean annual inflow (m^3^/s), k_y_ = S_beg_/αC and c = C/I_a_. S_beg_ is the reservoir storage at the beginning of a year (m^3^), C is the maximum storage capacity of the reservoir (m^3^), I_a_ is the mean total annual inflow (m^3^/yr), α is an empirical coefficient (0.85 in this study, as suggested by Hanasaki *et al*.^12^ based on sensitivity analysis), which influences inter-annual variation in releases.

The gross storage capacity (billion cubic meters: BCM) of the reservoirs, height (m), total installed capacity (MW) of the dams, and reservoir storage (2002–2014 period) were obtained from India WRIS (www.india-wris.nrsc.gov.in) (Table S1). The variation in the storage of the reservoirs is governed by the inflow from the upstream catchment area and outflow from the reservoir to meet the purpose of the dams (hydropower generation, irrigation, and other water demands). Inflow to the reservoir is primarily influenced by the precipitation over the catchment area (Fig. S13). The seasonal storage variation was used to determine the operation year for each reservoir, which starts from September (once the monsoon season is over), based on the criteria that the reservoir begins releasing water from this month^12^. The simulated streamflow was fed to a reservoir as inflow and the other parameters of the dam to estimate the monthly release were obtained using the Eq. (1). Two conditions were enforced while using the method:

- The minimum monthly release was set as 10% of the monthly inflow to the reservoir, and
- As mentioned in Liu *et al*.^5^, a minimum of 10% of the total capacity of reservoirs is maintained.

The monthly storage of the reservoir was calculated using the monthly inflow and release, which was compared with the observed reservoir storage to ensure that storage follows the rule curve of each reservoir. Since we used the no irrigation demands case of the Hanasaki *et* *al*.^12^, the storage for each reservoir was higher than that from the observed rule curve of the reservoir. To meet the other requirements (industrial, residential, and irrigation water demands), surplus storage was deducted (for the other water demands), and the rule curve was followed (Fig. 2A). The developed hydropower (HP, kW) potential of a reservoir was then estimated based on the monthly release: HP = min(R_m_ × h × g, IHC). Hydraulic head (h) was assumed to be linearly related to the reservoir storage, h = S/A, where S is the mean reservoir storage during the estimation time step, and A is the reservoir area. Also, A = C/H, where H is the height of the dam, which is also the maximum of h.

## Electronic supplementary material

Supplementary Material

## Data Availability

All the data used in this study will be made available upon request to the corresponding author.
