# Supplementary material for: Projected Increase in Hydropower Production in India under Climate Change
Source: Sci Rep. 2018 Aug 20;8:12450. doi: 10.1038/s41598-018-30489-4 (PMC6102243; doi:10.1038/s41598-018-30489-4)
Supplement: Supplementary file 1 — Supplementary Material [file 41598_2018_30489_MOESM1_ESM.doc]

**Supplemental Information**

**Projected Increase in Hydropower Production in India under Climate Change**

Syed Azhar Ali, Saran Aadhar, Harsh Shah, and Vimal Mishra*

Civil Engineering, Indian Institute of Technology Gandhinagar, Gandhinagar, 382355, India

Corresponding author: vmishra@iitgn.ac.in,+91-9687944337


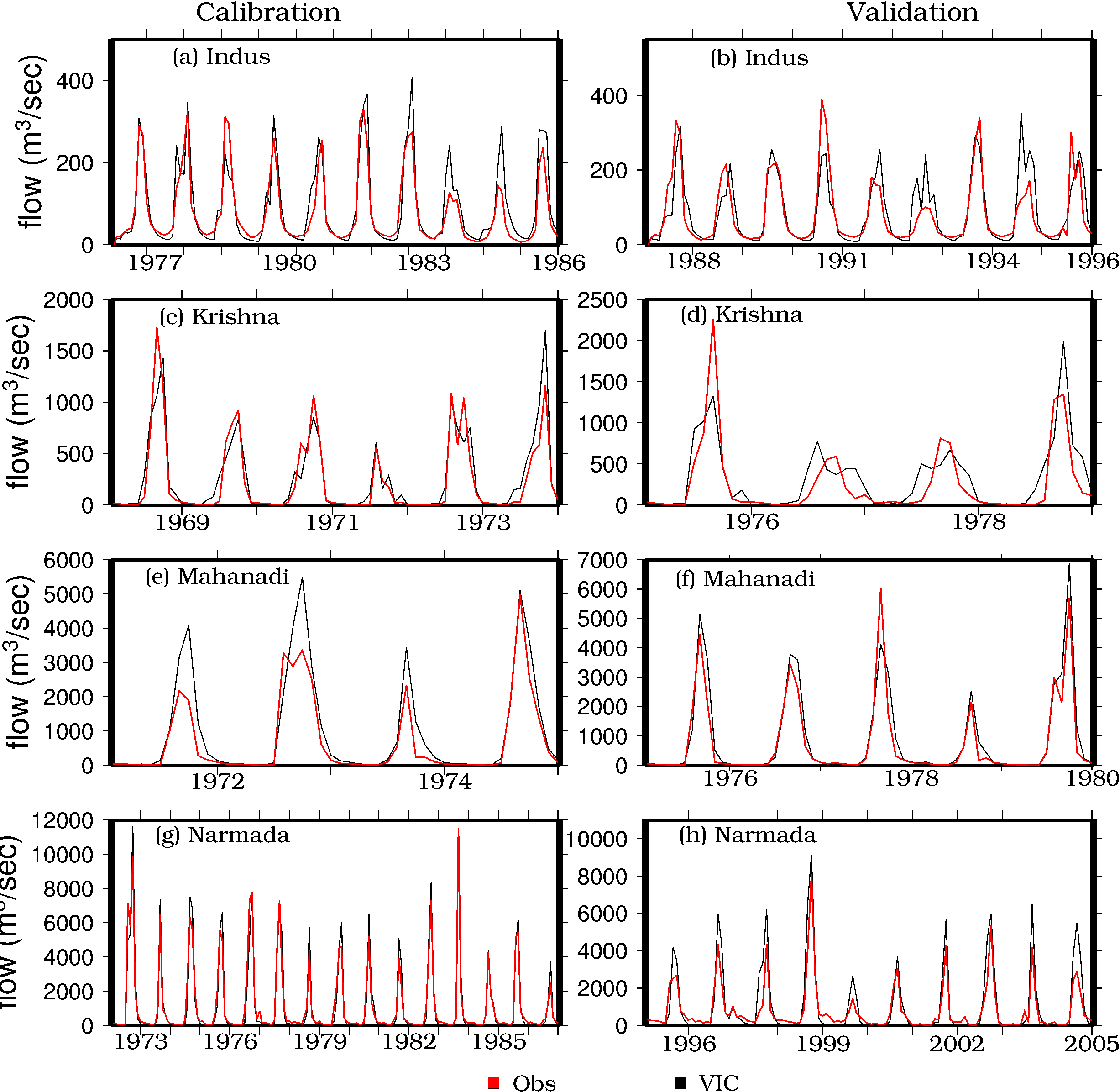


Figure S1 Monthly calibration and evaluation of the VIC model over Indus (a, b), Krishna (c, d), Mahanadi (e, f) and Narmada (g, h) river basins against observed streamflow. The figure was developed using the Generic Mapping Tools (GMT) version 5.4.2 (http://gmt.soest.hawaii.edu).


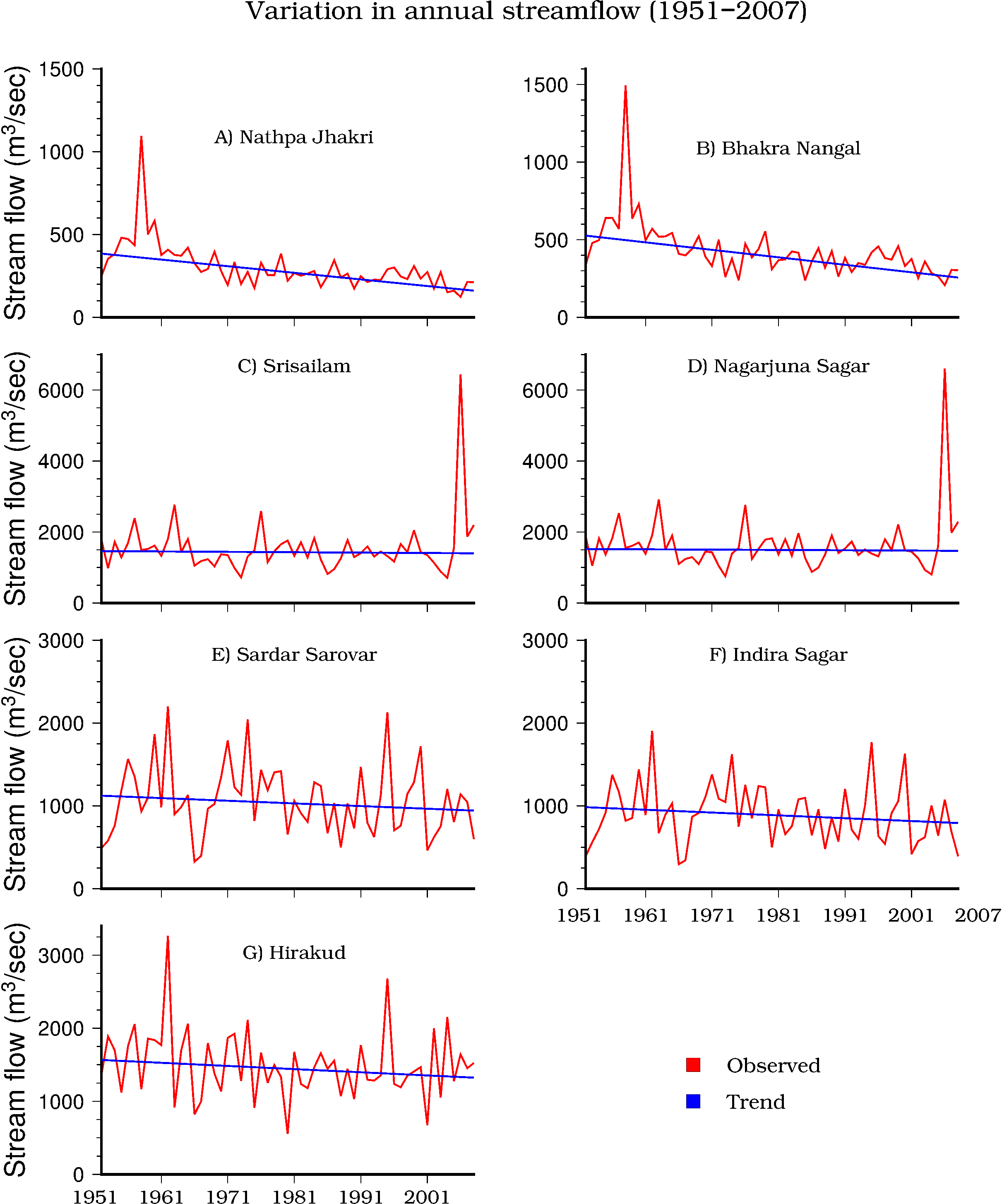


Figure S2 Annual streamflow variation and trend in the reservoirs A) Nathpa Jhakri, B) Bhakra Nangal, C) Srisailam, D) Nagarjuna Sagar, E) Sardar Sarovar, F) Indira Sagar, and G) Hirakud during 1951-2007. The figure was developed using the Generic Mapping Tools (GMT) version 5.4.2 ([**http://gmt.soest.hawaii.edu**](http://gmt.soest.hawaii.edu/)).


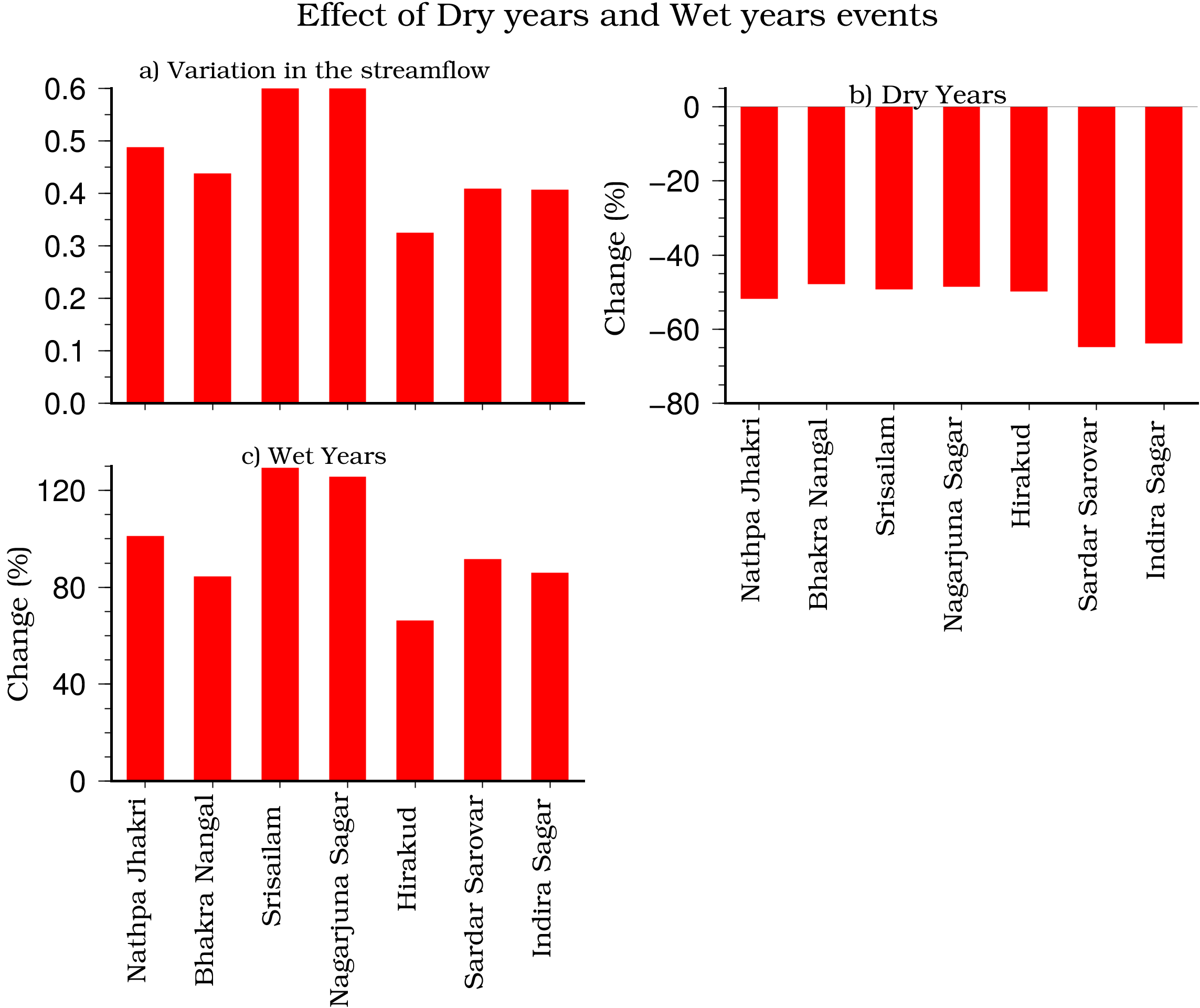


**Figure S3** (a) Coefficient of variation (CV) of the monsoon season streamflow simulated using the VIC model for the period of 1951-2007.Composite anomalies of the monsoon season streamflow for five extremes (b) Dry, and (c) Wet years (see Table S4 for more details). The figure was developed using the Generic Mapping Tools (GMT) version 5.4.2 (http://gmt.soest.hawaii.edu).


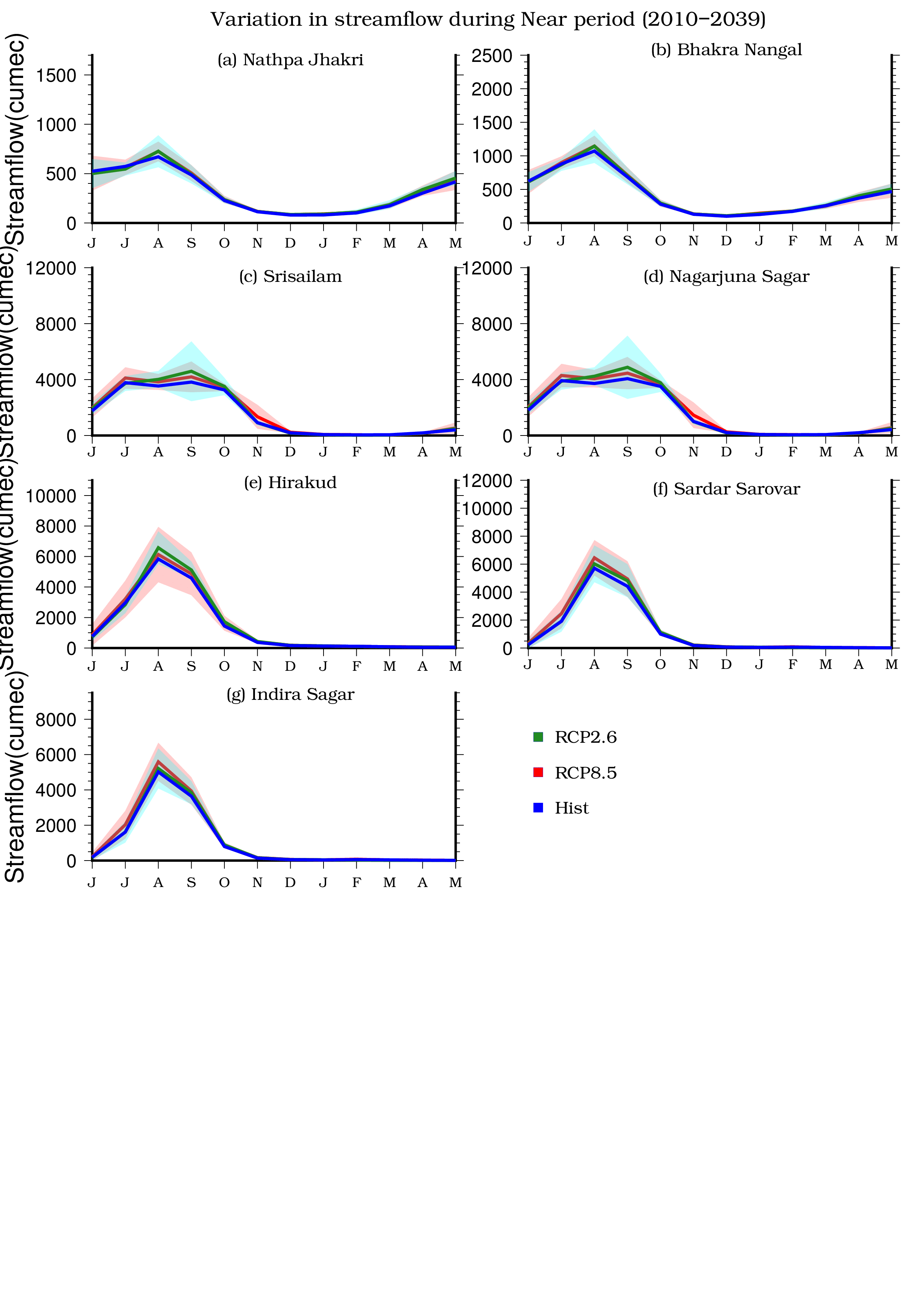


Figure S4 Projected streamflow (m^3^/sec) seasonal variation during historic period (1971-2000, blue) and near period (2010-2039) under RCP 2.6 (green) and RCP 8.5 (red) for a) Nathpa Jhakri, b) Bhakra Nangal, c) Srisailam, d) Nagarjuna Sagar, e) Hirakud, f) Sardar Sarovar, and g) Indira Sagar. Shaded regions show intermodel variability due to GCMs under RCP 2.6 (cyan) and RCP 8.5 (pink). The figure was developed using the Generic Mapping Tools (GMT) version 5.4.2 (http://gmt.soest.hawaii.edu).


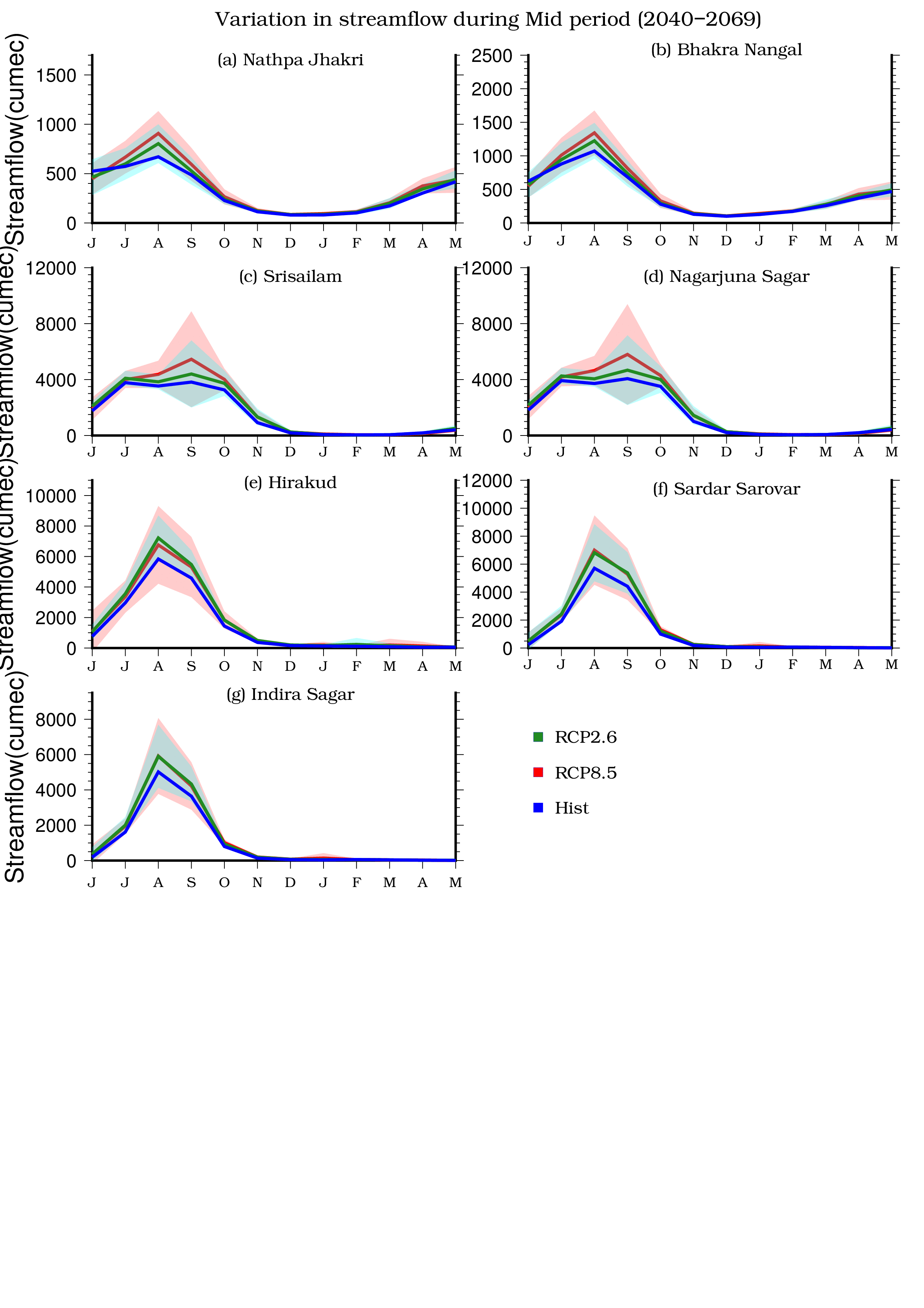


Figure S5 Same as Fig. S3 but for mid period (2040-2069). The figure was developed using the Generic Mapping Tools (GMT) version 5.4.2 (http://gmt.soest.hawaii.edu).


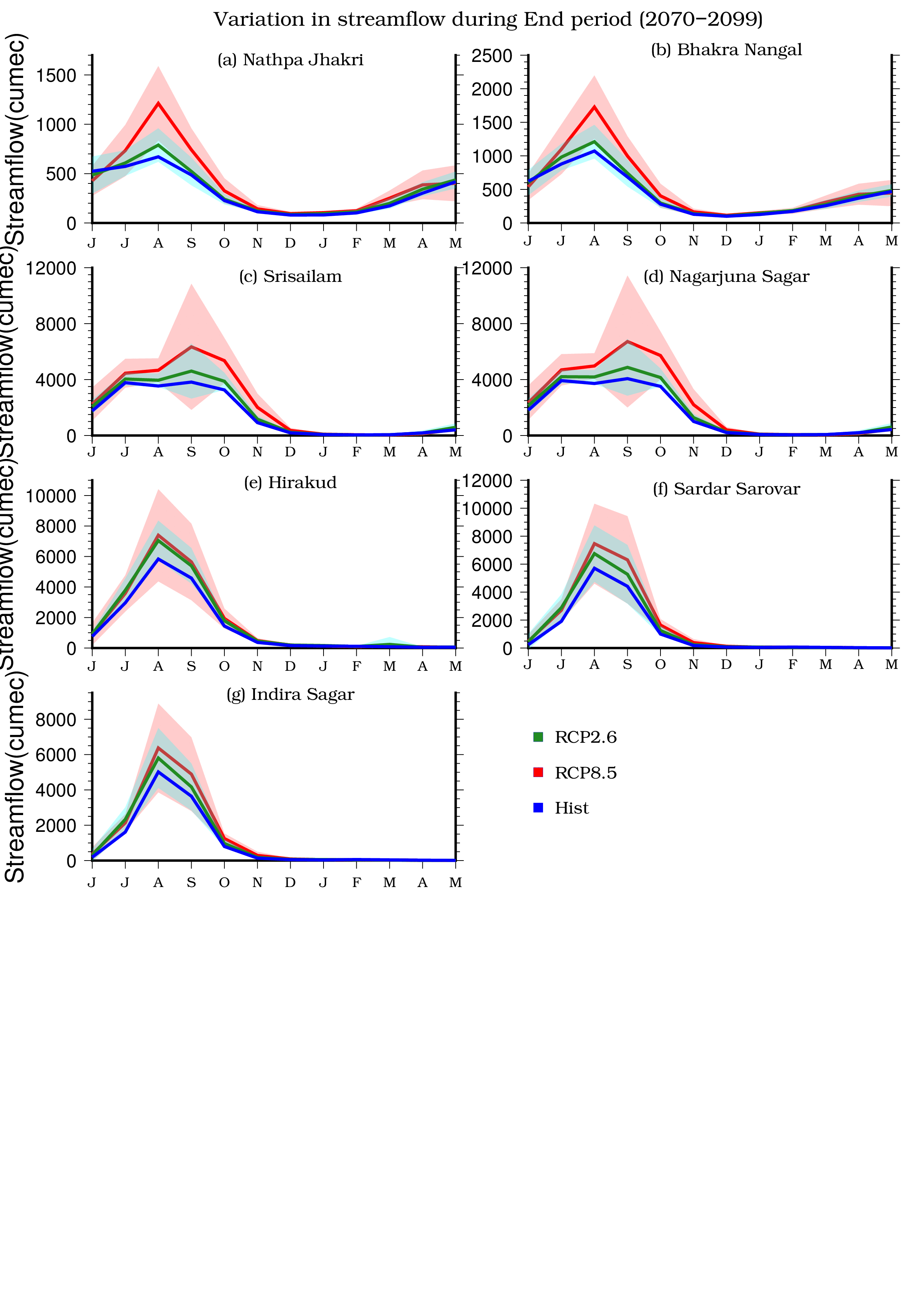


Figure S6 Same as Fig. S3 but for end period (2070-2099). The figure was developed using the Generic Mapping Tools (GMT) version 5.4.2 (http://gmt.soest.hawaii.edu).


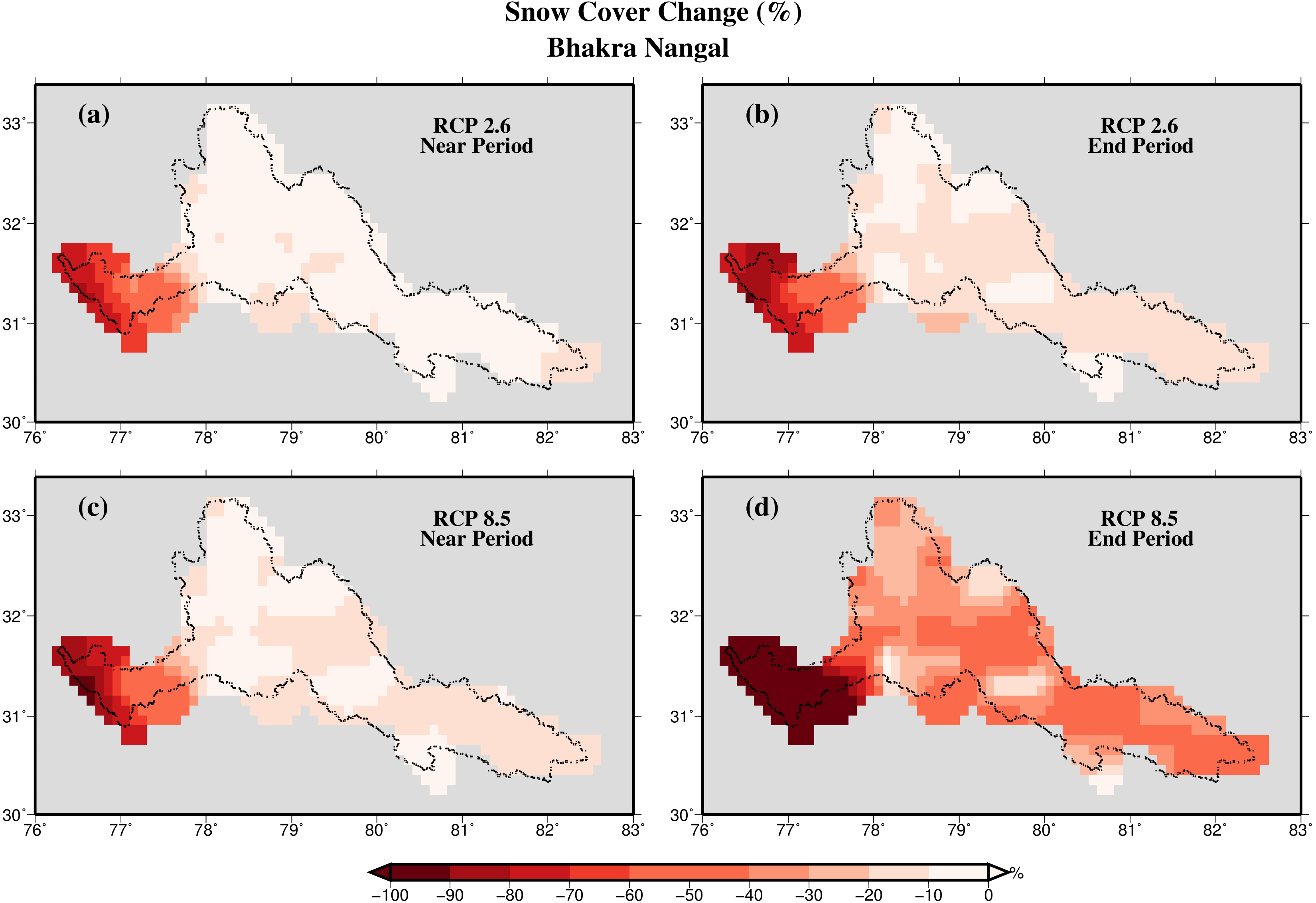


Figure S7 Projected % change in snow cover (mm/day) in (a-b) Bhakra Nangal and (c-d) Nathpa Jhakri reservoir basin during (a, c) near period (2010-2039) and (b, d) end period (2070-2099) under RCP 2.6 and RCP 8.5 with reference to the historical period (1971-2000). The figure was developed using the Generic Mapping Tools (GMT) version 5.4.2 (http://gmt.soest.hawaii.edu).


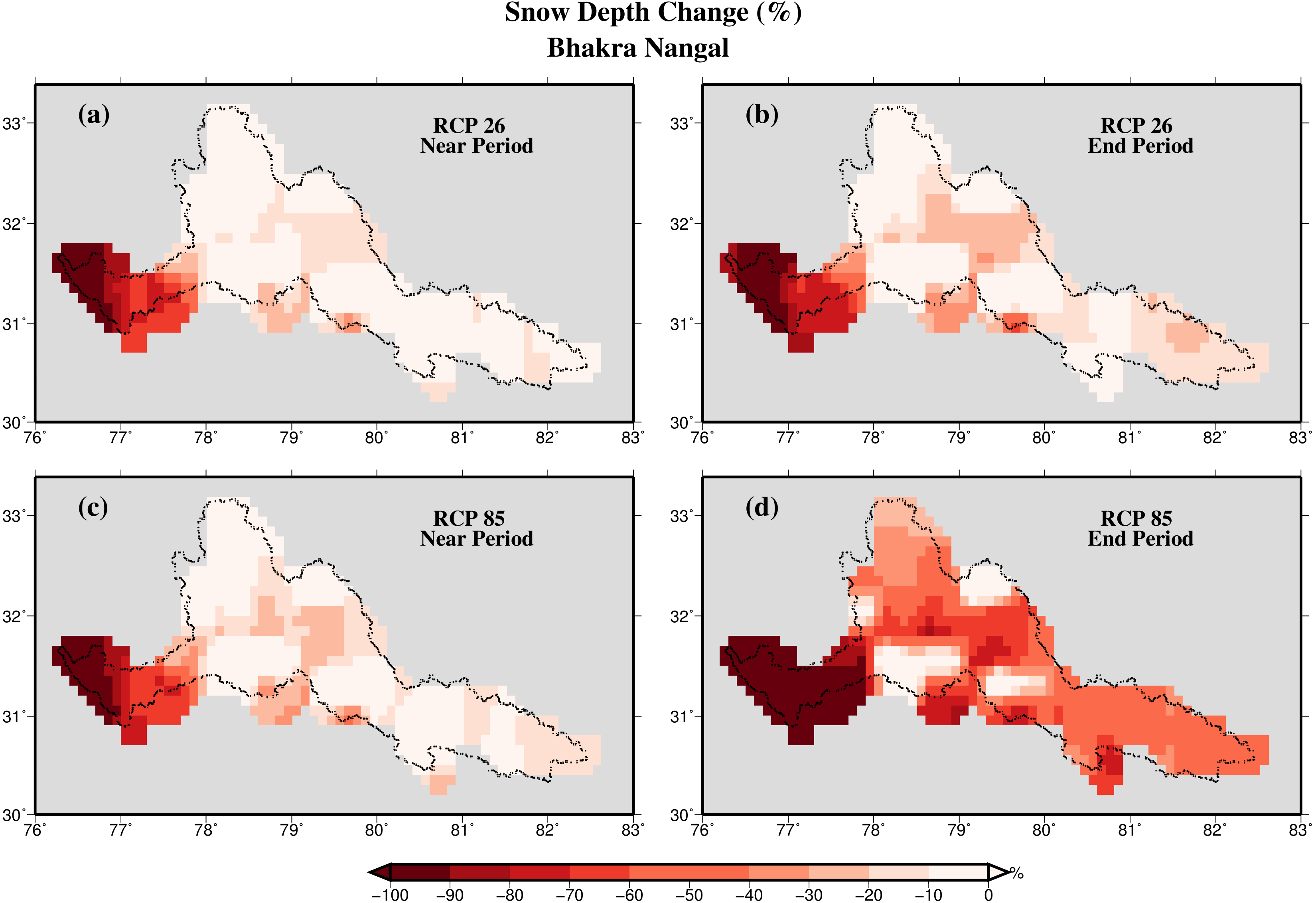


Figure S8 Same as figure S9 but for snow depth (mm/day). The figure was developed using the Generic Mapping Tools (GMT) version 5.4.2 (http://gmt.soest.hawaii.edu).


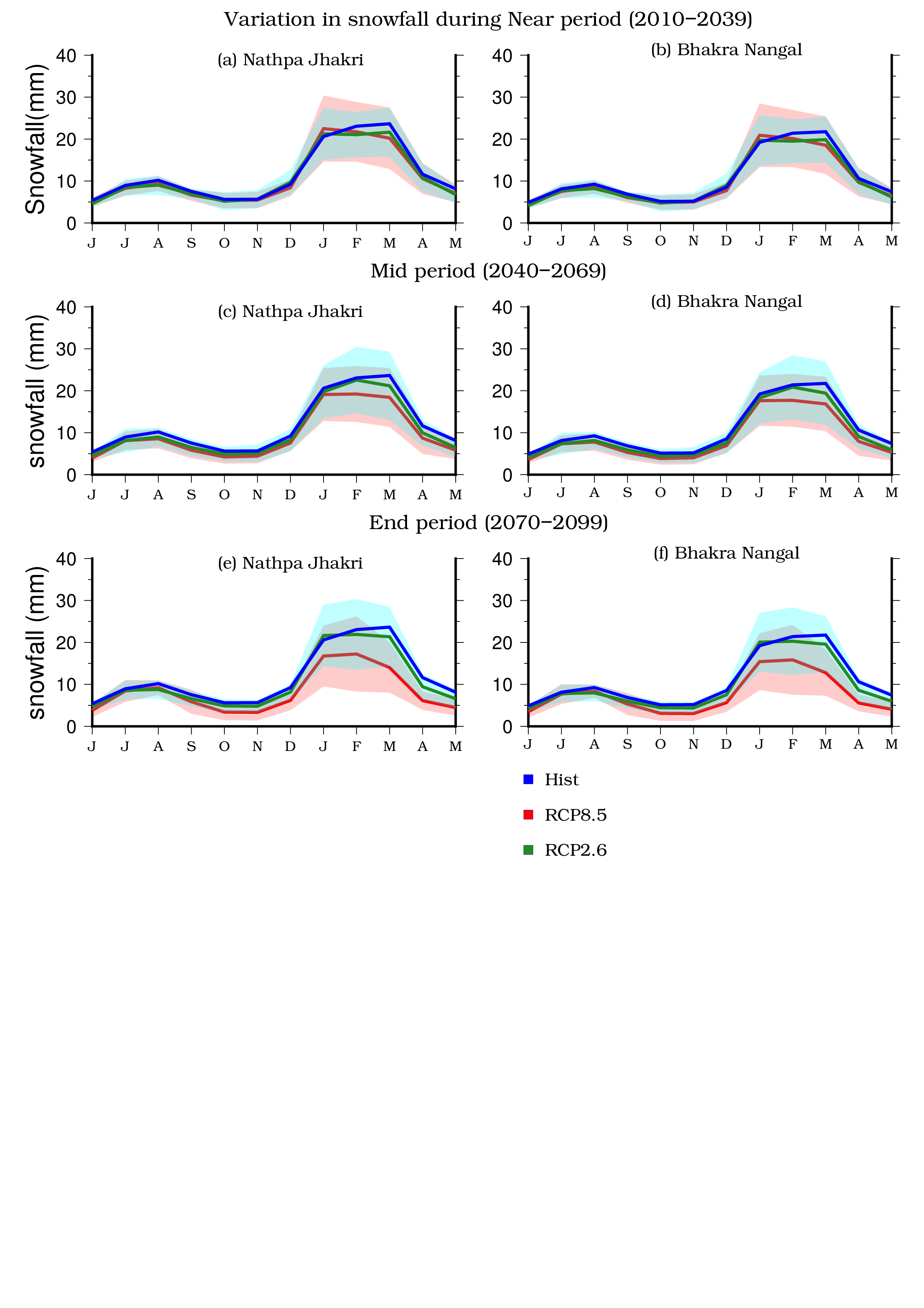


Figure S9 Multimodel ensemble mean projected change in snowfall under the projected future climate. The figure was developed using the Generic Mapping Tools (GMT) version 5.4.2 (http://gmt.soest.hawaii.edu).


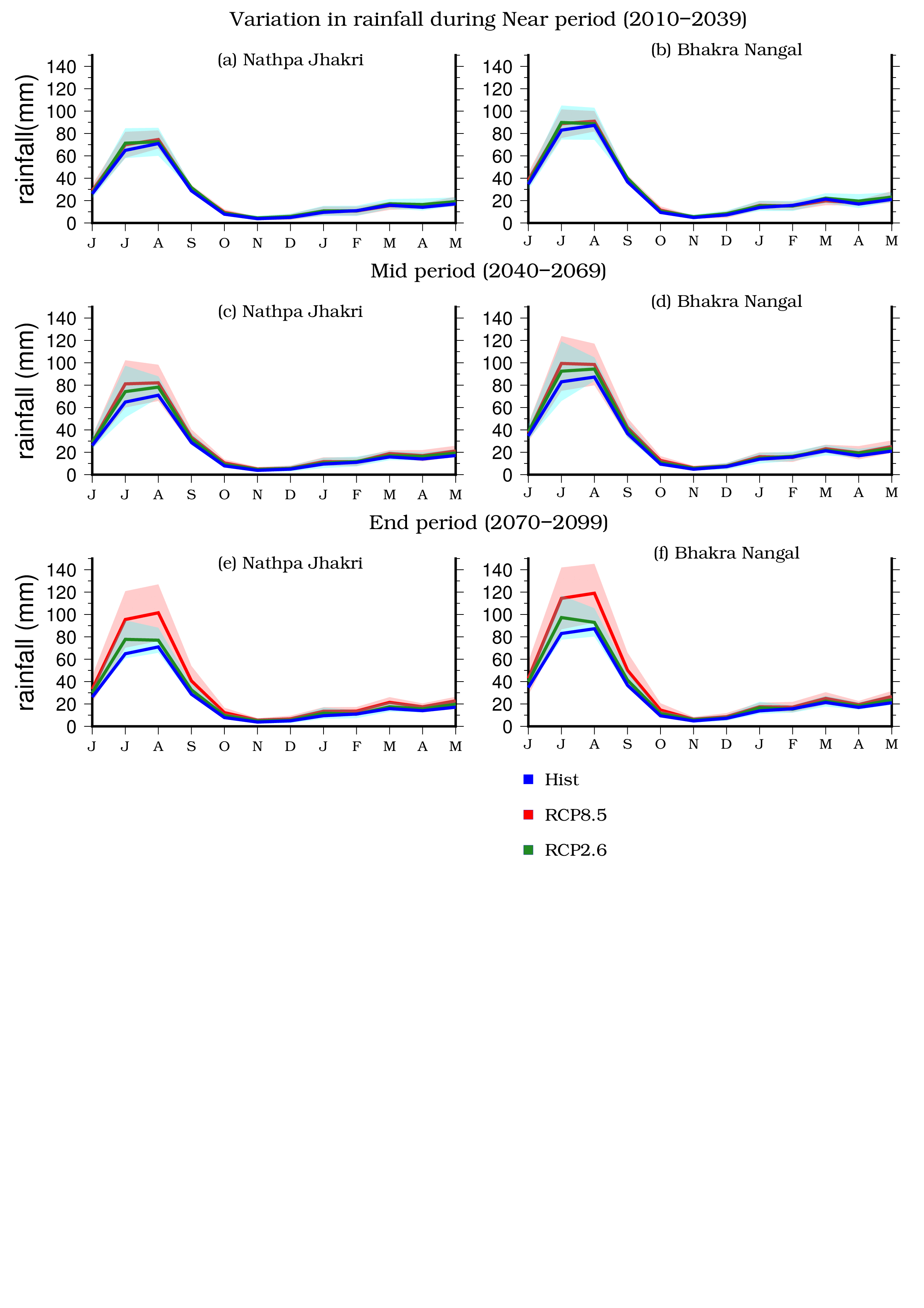


Figure S10 Multimodel ensemble mean projected change in rainfall under the projected future climate. The figure was developed using the Generic Mapping Tools (GMT) version 5.4.2 (http://gmt.soest.hawaii.edu).


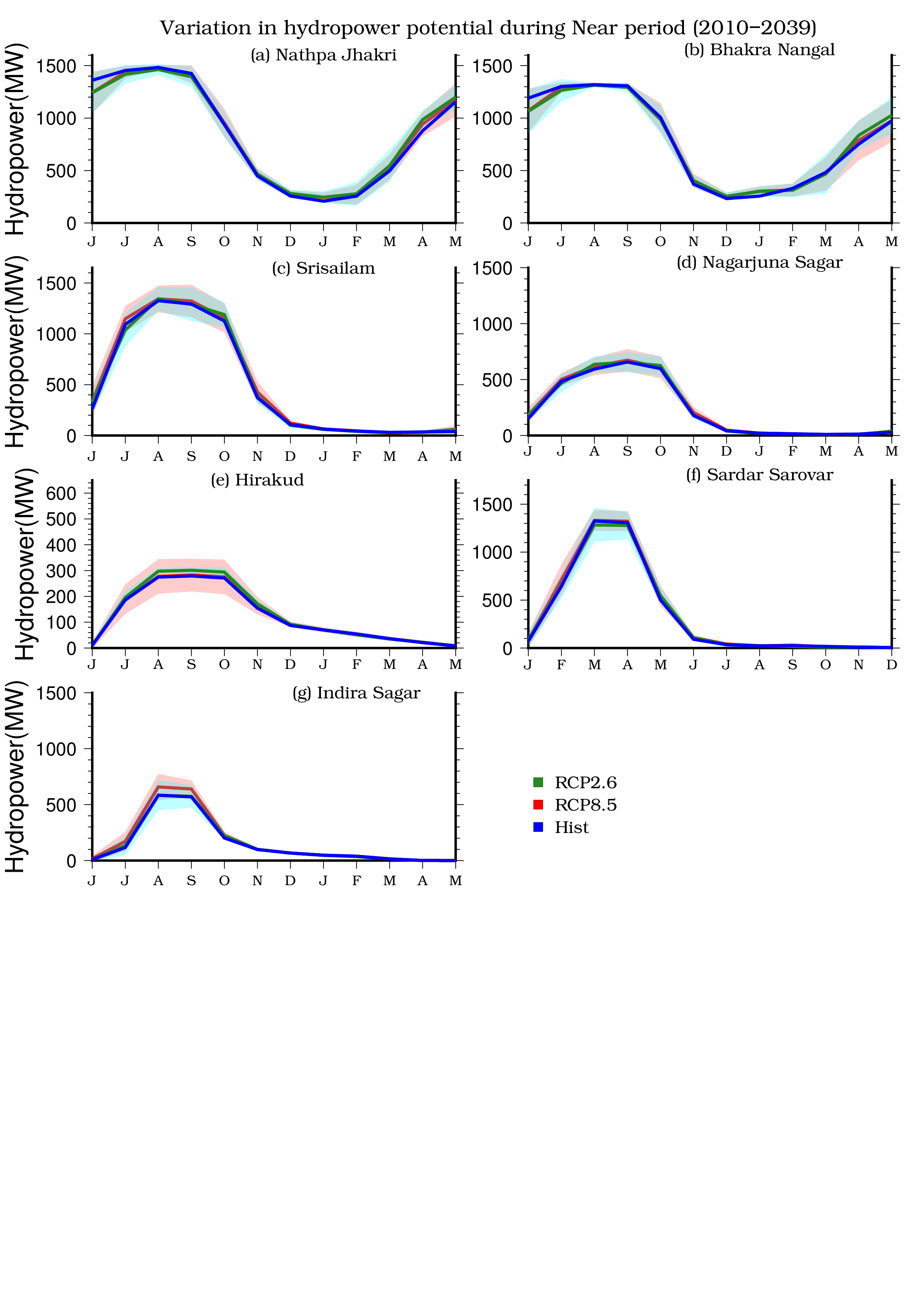


Figure S11 Same as Fig. 6 but for near period (2010-2039). The figure was developed using the Generic Mapping Tools (GMT) version 5.4.2 (http://gmt.soest.hawaii.edu).


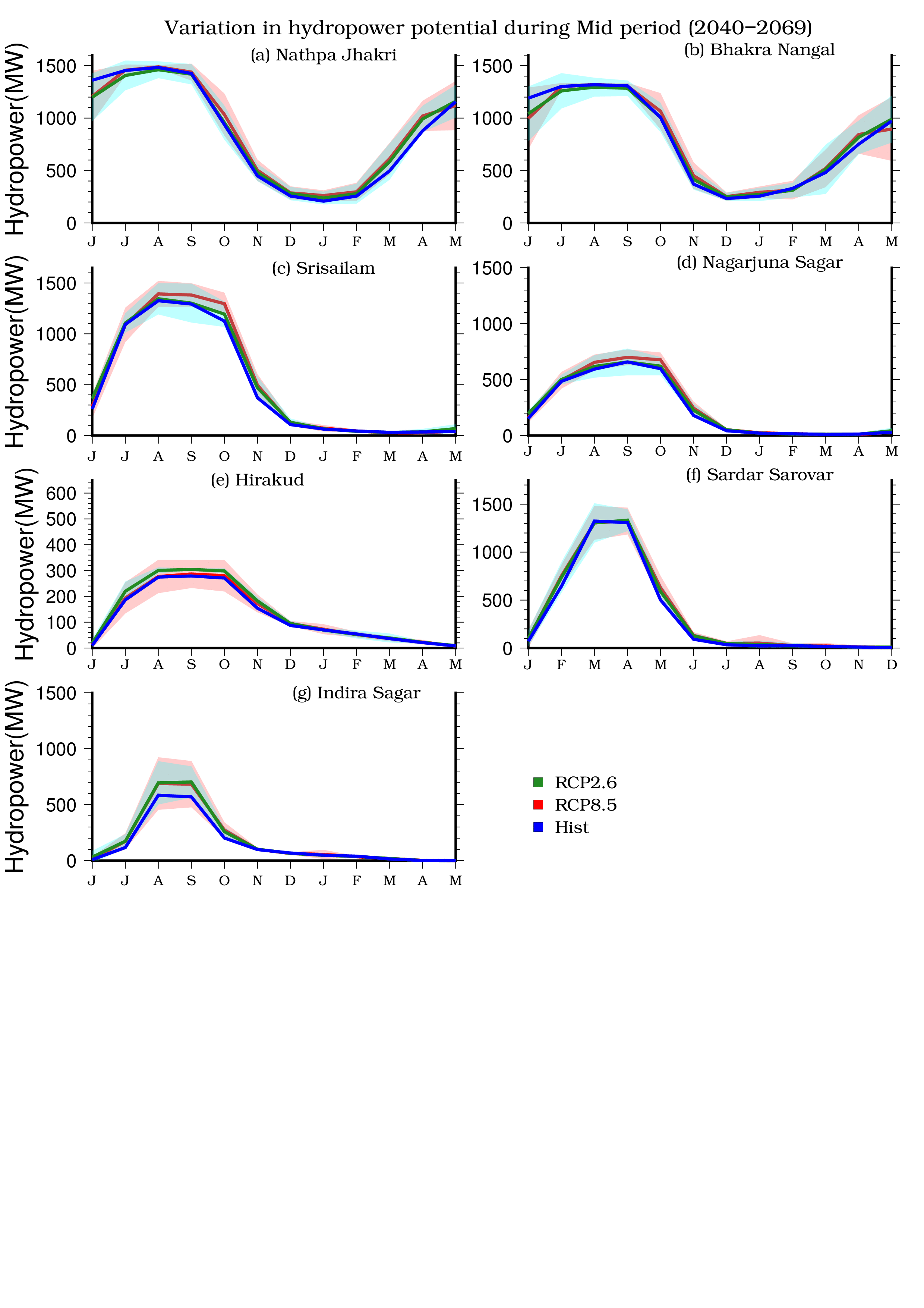


Figure S12 Same as Fig. 6 but for mid period (2040-2069). The figure was developed using the Generic Mapping Tools (GMT) version 5.4.2 (http://gmt.soest.hawaii.edu).


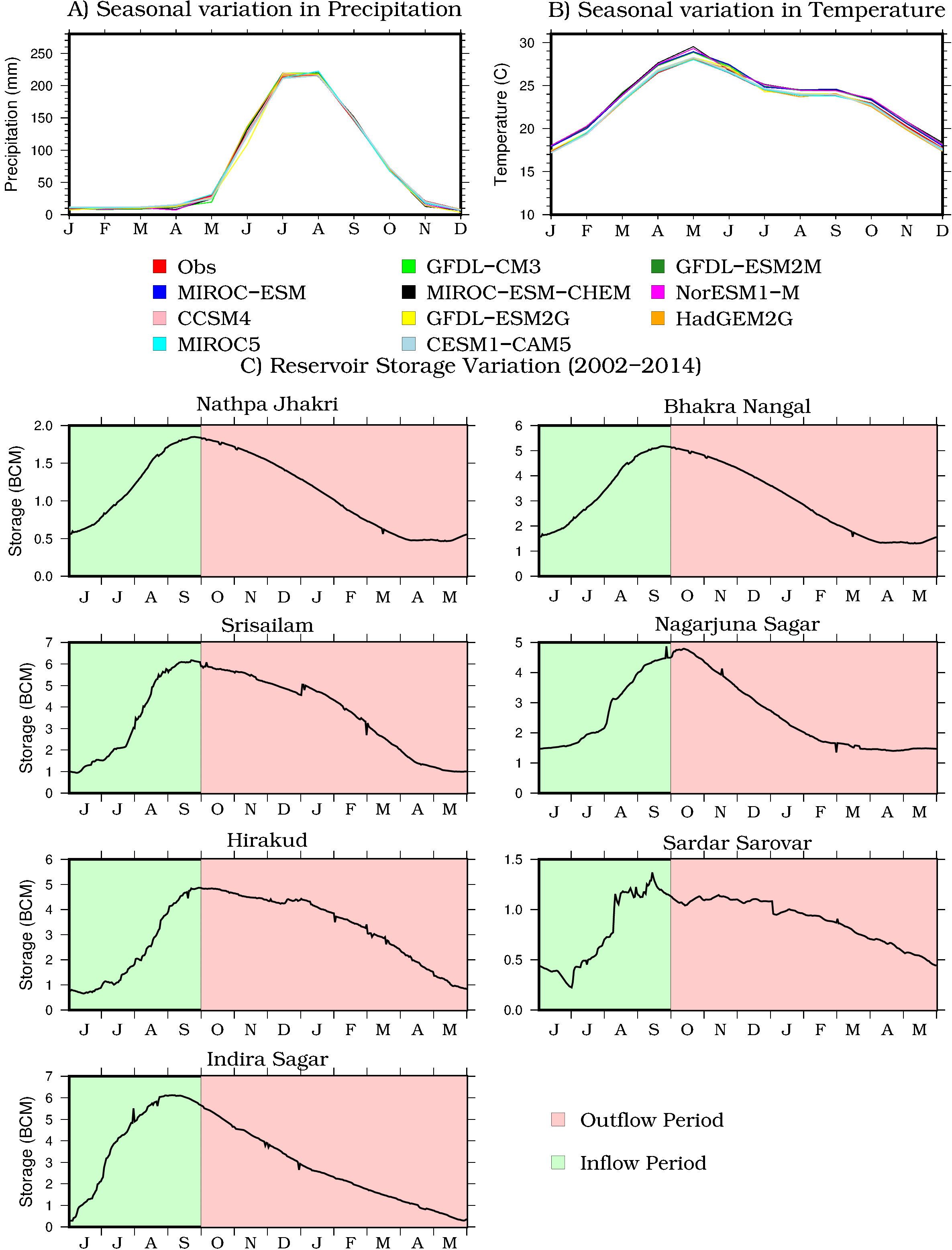


Figure S13 Mean monthly change in (A) Precipitation and (B) Temperature in observed and downscaled 5 GCMs data during historic period (1971-2000). Average daily variation in the storage (C) of the reservoirs for inflow period (June- September, green) and outflow period (October – May, pink) during the period 2002-2014. The figure was developed using the Generic Mapping Tools (GMT) version 5.4.2 (http://gmt.soest.hawaii.edu).

Table S1: Details of the Indian Sub-continental basins along with the gage stations (Source – Derived from India WRIS)

| **Dam** | **Latitude** | **Longitude** | **Height (m)** | **Gross Storage Capacity (BCM)** | **Total Installed Capacity (MW)** | **Basin** |
| --- | --- | --- | --- | --- | --- | --- |
| ***Nathpa Jhakri*** | 31.5639 | 77.9803 | 62.5 | 3.43 | 1500 | Indus |
| ***Bhakra Nangal*** | 31.4108 | 76.4333 | 167.64 | 9.86 | 1325 | Indus |
| ***Srisailam*** | 16.0869 | 78.8972 | 145 | 8.72 | 1670 | Krishna |
| ***Nagarjuna Sagar*** | 16.5756 | 79.3117 | 124.66 | 11.55 | 816 | Krishna |
| ***Hirakud*** | 21.57 | 83.87 | 60.96 | 8.10 | 307.5 | Mahanadi |
| ***Sardar Sarovar*** | 21.8303 | 73.7472 | 163 | 1.54 | 1450 | Narmada |
| ***Indira Sagar*** | 22.2839 | 76.4714 | 91.4 | 12.2 | 1000 | Narmada |

Table S2: Details of the Indian Sub-continental basins along with the gage stations (Source – Derived from India WRIS)

| **S. No.** | **Indian Sub-continental Basin** | **Catchment Area (Km^2^)** | **Gauging Station** | | |
| --- | --- | --- | --- | --- | --- |
|  |  |  | **Name** | **Latitude** | **Longitude** |
| 1 | **Indus** | 1116875 | Khab | 31.7999 | 78.6441 |
| 2 | **Krishna** | 282500 | Takli | 17.4141 | 75.8475 |
| 3 | **Mahanadi** | 157500 | Basantpur | 21.7219 | 82.7894 |
| 4 | **Narmada** | 128125 | Garudeshwar | 21.8869 | 73.6455 |

**Table S3:** Trends in observed mean annual precipitation, air temperature and model simulated streamflow and corresponding p-values for the period of 1951-2007. (* indicate values at 5% significance level or 95% confidence interval)

| Reservoir | Precipitation | | Temperature | | Streamflow | |
| --- | --- | --- | --- | --- | --- | --- |
|  | Trend (mm/year) | p-value | Trend (ºC/year) | p-value | Trend (m3/s/year) | p-value |
| Nathpa Jhakri | -4.35* | 0.0000 | 0.0093 | 0.16 | -3.99* | 0.0000 |
| Bhakhara Nangal | -4.35* | 0.0000 | 0.0093 | 0.16 | -4.82* | 0.0000 |
| Srisailam | -0.78 | 0.49 | 0.009* | 0.0003 | -1.1 | 0.74 |
| Nagarujana Sagar | -0.87 | 0.43 | 0.01* | 0.0000 | -0.91 | 0.78 |
| Hirakud | -1.11 | 0.32 | 0.0053* | 0.0003 | -4.32 | 0.26 |
| Sardar Sarovar | -1.11 | 0.47 | 0.0078* | 0.0007 | -3.21 | 0.40 |
| Indira Sagar | -1.36 | 0.23 | 0.007* | 0.0023 | -3.41 | 0.27 |

Table S4: List of years of extreme Dry and Wet year events for the reservoirs during the period 1951-2007.

| Events | **Nathpa Jhakri** | **Bhakra Nangal** | **Srisailam** | **Nagarjuna Sagar** | **Hirakud** | **Sardar Sarovar** | **Indira Sagar** |
| --- | --- | --- | --- | --- | --- | --- | --- |
| **Dry years** | 2005 | 2005 | 2003 | 1972 | 1979 | 1951 | 1951 |
|  | 1974 | 1984 | 1972 | 2003 | 2000 | 1965 | 1965 |
|  | 2004 | 1974 | 1985 | 1985 | 1965 | 1966 | 1966 |
|  | 1984 | 1972 | 1952 | 1952 | 1962 | 1987 | 2007 |
|  | 2003 | 1989 | 2002 | 2002 | 1974 | 2000 | 2000 |
| **Wet Years** | 1964 | 1961 | 1983 | 1988 | 1971 | 1970 | 1959 |
|  | 1968 | 1968 | 2006 | 2006 | 1964 | 1959 | 1999 |
|  | 1958 | 1958 | 2007 | 2007 | 2001 | 1973 | 1973 |
|  | 1959 | 1959 | 1962 | 1962 | 1994 | 1961 | 1961 |
|  | 1957 | 1957 | 2005 | 2005 | 1961 | 1994 | 1994 |

**Table S5:** Changes and intermodel variation (standard deviation) in precipitation, air temperature, streamflow, and hydropower potential under RCP 2.6, 4.5, 6.0, and 8.5 for the Near (2010-2039), Mid (2040-2069), and End (2070-2099).

| **A) Nathpa Jhakri** | | | | |
| --- | --- | --- | --- | --- |
|  | **RCP 2.6** | | **RCP 8.5** | |
|  | **Mean** | **Std** | **Mean** | **Std** |
|  | **Precipitation (%)** | | | |
| **Near** | 3.04 | 6.48 | 2.75 | 6.74 |
| **Mid** | 4.77 | 11.98 | 6.74 | 13.02 |
| **End** | 6.36 | 11.53 | 17.04 | 20.76 |
|  | **Temperature (^o^C)** | | | |
| **Near** | 1.40 | 0.40 | 1.64 | 0.20 |
| **Mid** | 2.10 | 0.86 | 3.73 | 0.80 |
| **End** | 2.06 | 0.95 | 6.32 | 1.62 |
|  | **Streamflow (%)** | | | |
| **Near** | 7.71 | 9.13 | 7.04 | 9.55 |
| **Mid** | 11.60 | 18.80 | 18.41 | 22.72 |
| **End** | 13.45 | 18.85 | 38.31 | 35.31 |
|  | **Hydropower (%)** | | | |
| **Near** | 2.75 | 5.31 | 2.21 | 7.17 |
| **Mid** | 2.84 | 10.64 | 4.37 | 10.25 |
| **End** | 4.52 | 9.97 | 8.33 | 13.30 |

| **B) Bhakra Nangal** | | | | |
| --- | --- | --- | --- | --- |
|  | **RCP 2.6** | | **RCP 8.5** | |
|  | **Mean** | **Std** | **Mean** | **Std** |
|  | **Precipitation (%)** | | | |
| **Near** | 2.95 | 6.09 | 2.76 | 6.37 |
| **Mid** | 4.38 | 11.73 | 5.95 | 12.80 |
| **End** | 6.18 | 11.17 | 15.30 | 20.16 |
|  | **Temperature (^o^C)** | | | |
| **Near** | 1.38 | 0.39 | 1.63 | 0.22 |
| **Mid** | 2.07 | 0.82 | 3.68 | 0.79 |
| **End** | 2.02 | 0.92 | 6.25 | 1.58 |
|  | **Streamflow (%)** | | | |
| **Near** | 6.64 | 8.21 | 6.24 | 9.03 |
| **Mid** | 9.52 | 18.00 | 14.33 | 21.80 |
| **End** | 12.05 | 17.68 | 30.98 | 33.50 |
|  | **Hydropower (%)** | | | |
| **Near** | 2.14 | 5.45 | 0.82 | 8.58 |
| **Mid** | 0.87 | 10.86 | 0.61 | 9.24 |
| **End** | 2.93 | 10.97 | 3.47 | 12.77 |

| **C) Srisailam** | | | | |
| --- | --- | --- | --- | --- |
|  | **RCP 2.6** | | **RCP 8.5** | |
|  | **Mean** | **Std** | **Mean** | **Std** |
|  | **Precipitation (%)** | | | |
| **Near** | 2.93 | 5.40 | 3.93 | 4.74 |
| **Mid** | 5.96 | 8.77 | 8.59 | 7.27 |
| **End** | 8.14 | 4.89 | 18.30 | 14.61 |
|  | **Temperature (^o^C)** | | | |
| **Near** | 0.82 | 0.18 | 0.93 | 0.15 |
| **Mid** | 1.15 | 0.31 | 2.24 | 0.36 |
| **End** | 1.22 | 0.40 | 3.82 | 0.78 |
|  | **Streamflow (%)** | | | |
| **Near** | 8.50 | 10.78 | 10.39 | 12.54 |
| **Mid** | 13.44 | 16.47 | 21.67 | 18.11 |
| **End** | 15.33 | 9.53 | 43.99 | 35.85 |
|  | **Hydropower (%)** | | | |
| **Near** | 2.41 | 4.64 | 5.28 | 6.41 |
| **Mid** | 7.17 | 9.26 | 8.64 | 8.02 |
| **End** | 9.02 | 5.51 | 14.86 | 14.49 |

| **D) Nagarjuna Sagar** | | | | |
| --- | --- | --- | --- | --- |
|  | **RCP 2.6** | | **RCP 8.5** | |
|  | **Mean** | **Std** | **Mean** | **Std** |
|  | **Precipitation (%)** | | | |
| **Near** | 2.91 | 5.31 | 3.97 | 4.69 |
| **Mid** | 5.94 | 8.62 | 8.66 | 7.15 |
| **End** | 8.05 | 4.70 | 18.48 | 14.71 |
|  | **Temperature (^o^C)** | | | |
| **Near** | 0.82 | 0.18 | 0.93 | 0.15 |
| **Mid** | 1.15 | 0.32 | 2.25 | 0.37 |
| **End** | 1.22 | 0.41 | 3.82 | 0.79 |
|  | **Streamflow (%)** | | | |
| **Near** | 8.46 | 10.56 | 10.55 | 12.30 |
| **Mid** | 13.38 | 16.04 | 22.09 | 18.00 |
| **End** | 15.23 | 9.00 | 44.75 | 35.90 |
|  | **Hydropower (%)** | | | |
| **Near** | 3.90 | 6.62 | 5.13 | 5.85 |
| **Mid** | 7.42 | 11.67 | 10.29 | 7.63 |
| **End** | 9.50 | 5.87 | 17.01 | 15.07 |

| **E) Hirakud** | | | | |
| --- | --- | --- | --- | --- |
|  | **RCP 2.6** | | **RCP 8.5** | |
|  | **Mean** | **Std** | **Mean** | **Std** |
|  | **Precipitation (%)** | | | |
| **Near** | 0.42 | 4.90 | 3.34 | 3.59 |
| **Mid** | 6.51 | 7.50 | 8.18 | 11.11 |
| **End** | 7.21 | 4.61 | 13.16 | 14.29 |
|  | **Temperature (^o^C)** | | | |
| **Near** | 0.94 | 0.25 | 1.05 | 0.16 |
| **Mid** | 1.19 | 0.45 | 2.65 | 0.40 |
| **End** | 1.28 | 0.49 | 4.45 | 0.68 |
|  | **Streamflow (%)** | | | |
| **Near** | 1.73 | 9.75 | 6.73 | 6.23 |
| **Mid** | 15.10 | 12.88 | 18.36 | 17.28 |
| **End** | 13.62 | 8.93 | 25.30 | 22.76 |
|  | **Hydropower (%)** | | | |
| **Near** | 0.42 | 4.00 | 2.45 | 4.83 |
| **Mid** | 4.67 | 6.79 | 5.11 | 7.39 |
| **End** | 3.86 | 3.57 | 8.16 | 18.71 |

| **F) Sardar Sarovar** | | | | |
| --- | --- | --- | --- | --- |
|  | **RCP 2.6** | | **RCP 8.5** | |
|  | **Mean** | **Std** | **Mean** | **Std** |
|  | **Precipitation (%)** | | | |
| **Near** | 2.86 | 5.08 | 6.45 | 4.82 |
| **Mid** | 10.13 | 10.49 | 10.23 | 13.96 |
| **End** | 11.61 | 7.55 | 17.69 | 17.77 |
|  | **Temperature (^o^C)** | | | |
| **Near** | 1.07 | 0.26 | 1.02 | 0.13 |
| **Mid** | 1.29 | 0.35 | 2.48 | 0.34 |
| **End** | 1.30 | 0.45 | 4.25 | 0.67 |
|  | **Streamflow (%)** | | | |
| **Near** | 6.36 | 10.05 | 15.16 | 9.96 |
| **Mid** | 22.93 | 18.11 | 24.18 | 25.36 |
| **End** | 24.84 | 15.02 | 39.93 | 32.22 |
|  | **Hydropower (%)** | | | |
| **Near** | 0.99 | 5.86 | 4.75 | 7.48 |
| **Mid** | 8.84 | 13.55 | 9.61 | 13.39 |
| **End** | 10.05 | 8.27 | 12.38 | 17.39 |

| **G) Indira Sagar** | | | | |
| --- | --- | --- | --- | --- |
|  | **RCP 2.6** | | **RCP 8.5** | |
|  | **Mean** | **Std** | **Mean** | **Std** |
|  | **Precipitation (%)** | | | |
| **Near** | 2.06 | 5.68 | 5.53 | 5.15 |
| **Mid** | 9.56 | 10.11 | 9.04 | 15.01 |
| **End** | 10.26 | 8.00 | 15.75 | 17.64 |
|  | **Temperature (^o^C)** | | | |
| **Near** | 1.10 | 0.29 | 1.05 | 0.16 |
| **Mid** | 1.31 | 0.36 | 2.52 | 0.34 |
| **End** | 1.34 | 0.46 | 4.33 | 0.63 |
|  | **Streamflow (%)** | | | |
| **Near** | 4.18 | 11.05 | 12.34 | 9.85 |
| **Mid** | 20.15 | 17.32 | 19.78 | 26.80 |
| **End** | 20.39 | 15.53 | 32.99 | 31.01 |
|  | **Hydropower (%)** | | | |
| **Near** | 2.87 | 12.45 | 13.75 | 12.12 |
| **Mid** | 23.28 | 20.99 | 20.53 | 28.76 |
| **End** | 23.30 | 14.43 | 25.46 | 29.18 |

Table S6: Details of the General Circulation Models (GCMs) data used

| **Model** | **Name** | **Developed by** | **Native resolution** | |
| --- | --- | --- | --- | --- |
|  |  |  | **Latitude** | **Longitude** |
| CCSM4 | Community Climate System Model version 4 | Climate and Global Dynamic Laboratory at NCAR | 0.9424 | 1.25 |
| CESM1-CAM5 | Community Atmosphere Model version 5 |  | 0.9424 | 1.25 |
| GFDL-CM3 | Geophysical Fluid Dynamics Laboratory-Coupled Model version 3 | Geophysical Fluid Dynamics Laboratory | 2 | 2.5 |
| GFDL-ESM2M | Geophysical Fluid Dynamics Laboratory-Earth System Model |  | 2.0225 | 2.5 |
| GFDL-ESM2G |  |  |  |  |
| HadGEM2-AO | Hadley Global Environment Model 2 – Atmosphere Ocean | National Institute of Meteorological Research (NIMAR), Korea Meteorological Administration and UK Met Office Hadley centre | 1.250 | 1.875 |
| MIROC5 | Model for Interdisciplinary Research on Climate version 5 | Japan Agency for Marine-Earth Science and Technology, Atmosphere and Ocean Research Institute (The University of Tokyo), and National Institute for Environmental Studies | 1.40 | 1.40625 |
| MIROC-ESM | Model for Interdisciplinary Research on Climate – Earth Surface Model |  | 2.7906 | 2.8125 |
| MIROC-ESM-CHEM | Model for Interdisciplinary Research on Climate – Earth Surface Model – Chemistry coupled |  | 2.7906 | 2.8125 |
| NorESM1-M | Norwegian Earth System Model 1 - medium resolution | Norwegian Climate Centre (NCC) | 1.8947 | 2.5 |

Table S7: Values of Nash Sutcliffe Efficiency (NE) and correlation coefficient (r) for comparison of hydrologic models simulated streamflow against observed streamflow at gage station location for the basins during the calibration period and validation period.

| **S. No.** | **Indian Sub-continental Basin** | **Gage Location** | **Calibration** | | | **Validation** | | |
| --- | --- | --- | --- | --- | --- | --- | --- | --- |
|  |  |  | **Duration** | **NE** | **r** | **Duration** | **NE** | **r** |
| 1 | Indus | Khab | 1977-1986 | 0.76 | 0.87 | 1988-1996 | 0.63 | 0.73 |
| 2 | Krishna | Takli | 1969-1974 | 0.82 | 0.91 | 1976-1979 | 0.64 | 0.82 |
| 3 | Mahanadi | Basantpur | 1972-1975 | 0.72 | 0.94 | 1976-1980 | 0.88 | 0.95 |
| 4 | Narmada | Garudeshwar | 1973-1987 | 0.94 | 0.97 | 1996-2005 | 0.66 | 0.95 |

1. Hempel, S., Frieler, K., Warszawski, L., Schewe, J. & Piontek, F. A trend-preserving bias correction &ndash; The ISI-MIP approach*. Earth Syst. Dy*n**.** 4, 219–236 (2013).
